# Effects of Gliadin consumption on the Intestinal Microbiota and Metabolic Homeostasis in Mice Fed a High-fat Diet

**DOI:** 10.1038/srep44613

**Published:** 2017-03-16

**Authors:** Li Zhang, Daniel Andersen, Henrik Munch Roager, Martin Iain Bahl, Camilla Hartmann Friis Hansen, Niels Banhos Danneskiold-Samsøe, Karsten Kristiansen, Ilinca Daria Radulescu, Christian Sina, Henrik Lauritz Frandsen, Axel Kornerup Hansen, Susanne Brix, Lars I. Hellgren, Tine Rask Licht

**Affiliations:** 1 National Food Institute, Technical University of Denmark, 2860 Søborg, Denmark; 2 Department of Veterinary Disease Biology, University of Copenhagen, 1871 Frederiksberg C, Denmark; 3 Department of Systems Biology, Technical University of Denmark, 2800 Kgs. Lyngby, Denmark; 4 Department of Biology, University of Copenhagen, 2100 København Ø, Denmark; 5 Department of Internal Medicine I, University Hospital of Schleswig-Holstein, Lübeck, Germany

## Abstract

Dietary gluten causes severe disorders like celiac disease in gluten-intolerant humans. However, currently understanding of its impact in tolerant individuals is limited. Our objective was to test whether gliadin, one of the detrimental parts of gluten, would impact the metabolic effects of an obesogenic diet. Mice were fed either a defined high-fat diet (HFD) containing 4% gliadin (n = 20), or a gliadin-free, isocaloric HFD (n = 20) for 23 weeks. Combined analysis of several parameters including insulin resistance, histology of liver and adipose tissue, intestinal microbiota in three gut compartments, gut barrier function, gene expression, urinary metabolites and immune profiles in intestinal, lymphoid, liver and adipose tissues was performed. Mice fed the gliadin-containing HFD displayed higher glycated hemoglobin and higher insulin resistance as evaluated by the homeostasis model assessment, more hepatic lipid accumulation and smaller adipocytes than mice fed the gliadin-free HFD. This was accompanied by alterations in the composition and activity of the gut microbiota, gut barrier function, urine metabolome, and immune phenotypes within liver and adipose tissue. Our results reveal that gliadin disturbs the intestinal environment and affects metabolic homeostasis in obese mice, suggesting a detrimental effect of gluten intake in gluten-tolerant subjects consuming a high-fat diet.

Gluten is the main structural protein complex in cereal seed endosperm, and is as such a natural component of flour-based bread, cakes, and pasta included in many Western diets. However, besides the recognized symptoms related to diagnosed gluten intolerance such as wheat allergy, celiac disease and nonceliac gluten sensitivity^1^, gluten may also hold disease-driving potentials in so-called gluten-tolerant individuals. This is especially evident in gluten elimination studies undertaken in subjects suffering from Irritable Bowel Syndrome, which report reduced bowel symptoms after short term intake of gluten-free diets^2^,^3^, but gluten-free diets may also have a beneficial effect on human type 1 diabetes^4^. The latter is supported by the observation that gluten increases incidences of type 1 diabetes in animal models^5^,^6^.

The metabolic effects of gluten in combination with a high-fat diet (HFD) is hitherto addressed in four animal studies. Two of these report marked detrimental effects of gluten intake on obesity and insulin resistance within eight weeks^7^,^8^, while two long-term studies show either no effects on these parameters or a fluctuating effect on glucose tolerance^9^,^10^. A study from 1978 reports that dietary gluten causes rearrangements of the plasma, liver and epididymal adipose tissue lipid pool in rats^11^. The causal mechanisms behind the effects of gluten in the context of a HFD however remain elusive, and explorative studies that map the interaction between the many involved host responses are thus highly needed to decipher the impact of gliadin in gluten-tolerant hosts.

Gluten is a heterogeneous compound based on prolamin and glutelin, and the prolamin fraction of wheat, gliadin, which contains peptides rich in glutamine and proline, is reported to play a key role in gluten intolerance^1^. The gliadin-derived proline-rich peptides are particularly resistant to proteolysis by digestive enzymes^12^, which means that gliadin peptides, including the gut-permeating peptides designated 111–130 and 151–170, the cytotoxic peptide 31–43, and the immune-modulating 33-mer peptide 57–89^13^, remain partly undigested and biologically active in the gastrointestinal tract. Activities of these peptides are not limited to induction of autoimmunity, but may also affect gluten-tolerant individuals^14^,^15^.

The gut microbiota interacts with host metabolism and immune system^16^,^17^, and thus also influences parameters related to metabolic syndrome^18^,^19^. Several bacteria isolated from the human gut are able to metabolise gluten^20^,^21^. Specifically, some strains of *Bifidobacterium* and *Lactobacillus* have been shown to hydrolyse gliadin peptides into inactive peptides, thereby counteracting gliadin-mediated effects on permeability^22^, inflammation^23^, and cell agglutination^24^. A change in gut microbiota composition and activity induced by gliadin consumption may therefore influence several factors of importance for host physiology. Nevertheless, the effects of gluten/gliadin intake on intestinal microbes in gluten-tolerant mice^5^,^9^or humans^25^,^26^ have been addressed only by very few studies, which were limited to ApoE-deficient mice, non-obese diabetic mice, and very small groups of healthy humans, and the results vary heavily. Therefore, effects of specific intervention with gliadin in gluten-tolerant models or subjects await comprehensive investigation.

Here, we aimed to comprehensively investigate the long-term effects of gliadin intake on host metabolic health and microbiota. Considering that animals with metabolic disorders are more susceptible to disturbance in metabolism, we chose HFD-fed mice as a sensitive model of gluten-tolerant, obese humans. We fed mice a synthetic diet with 60% of the energy originating from fat, and containing either 4% gliadin or no gliadin, for 23 weeks (Supplementary Table S1). We measured the effects on systemic host physiology, including glucose homeostasis, lipid metabolism and inflammation. Furthermore, we addressed whether and how these alterations were promoted by changes in specific host features including microbiota composition and activity, barrier function and immune responses within the gut, as well as the urinary metabolic signature and immune responses in liver and adipose tissue.

Our results demonstrate that gliadin affects both the intestinal microbiota and the ileal barrier function, and that consumption of this wheat component affects metabolic homeostasis as well as extra-intestinal immune responses in animals fed HFD. Importantly, explorative approaches and network analyses raise novel hypotheses about the underlying mechanisms behind effects of gliadin intake on metabolic health.

## Results

### Gliadin Intake Affected Glucose and Lipid Metabolic Homeostasis

**Figure 1:**
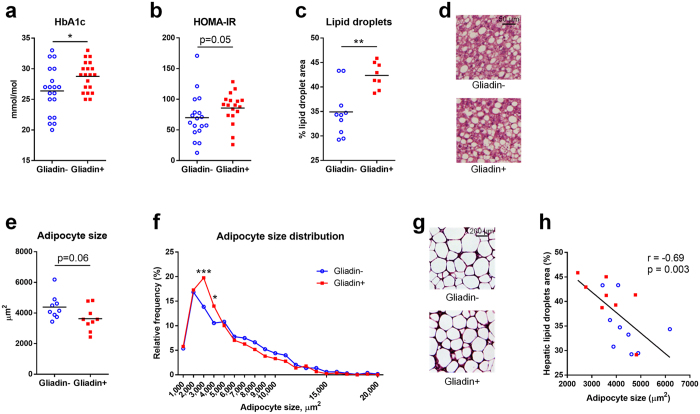
Gliadin affected glucose homeostasis, liver lipid accumulation and adipocyte size in eWAT. Mice were fed HFD (60% energy originating from fat) with gliadin (Gliadin+, n = 20) or without (Gliadin−, n = 19) for 23 weeks and subjected to measurements of metabolic features at termination. Panel (a) shows HbA1c levels in blood, (**b**) HOMA-IR, (**c**) percentage of lipid droplet area of the liver (each data point represents the average of six squares of 40,000 μm^2^), (**d**) hematoxylin- and eosin-stained hepatic sections, e the median adipocyte size of four different adipose tissue sections, (**f**) the relative frequency of the mean adipocyte size of four different adipose tissue sections, (**g**) hematoxylin- and eosin-stained eWAT sections, and h Spearman correlation between adipocyte size in eWAT and total lipid droplet area in the liver. In panels (a–c) and (e), horizontal lines represent the means, while asterisks represent statistically significant differences between the two feeding groups (*p < 0.05, **p < 0.01, unpaired t test or Mann-Whitney test). In panel (f), asterisks represent false discovery rate (FDR) corrected p values^71^ from multiple t tests (*q < 0.05, ***q < 0.001). In panel (h), the p value and r coefficient of Spearman correlation are listed, and the linear regression line is shown. In panels (c–h), n = 9–10 mice per group. See also Supplementary Fig. S1 and Supplementary Table S2.

After 23 weeks of HFD-based dietary intervention, Gliadin+ mice displayed significantly higher levels of glycated hemoglobin (HbA1c) than Gliadin- mice (Fig. 1a), suggesting a higher average blood glucose level in the Gliadin+ mice during the intervention period. Likewise, insulin resistance as determined by the Homeostasis Model Assessment of Insulin Resistance (HOMA-IR) was higher in the Gliadin+ group (Fig. 1b). Additionally, we observed marked differences in lipid storage. Gliadin+ mice exhibited more abundant lipid droplets in the liver (Fig. 1c and 1d), but smaller adipocyte size in the epididymal white adipose tissue (eWAT), also reflected in the adipocyte size distribution that revealed higher frequencies of small adipocytes of Gliadin+ mice in the range of 2,000–4,000 μm^2^ (Fig. 1e, 1f, 1g). Moreover, the adipocyte size and hepatic total lipid droplet area were strongly negatively correlated (Fig. 1h), suggesting a link between lipid storage capacity in the eWAT and lipid accumulation in the liver.

In spite of these differences, the Gliadin− and Gliadin+ mice had similar levels of many of the assessed metabolic parameters. Mice in both groups reacted similarly to a glucose challenge in oral glucose tolerance tests (OGTT), and showed impaired glucose clearance with increasing age and body weight (Supplementary Fig. S1). No differences were observed between the two groups with respect to fasting insulin levels, fasting glucose levels, or hepatic gene expression of enzymes involved in gluconeogenesis, glucose 6-phosphatase (*G6pase*) and phosphoenolpyruvate carboxykinase (*Pepck*, Supplementary Fig. S1). Additionally, there was no gliadin-induced difference in ileal expression of *Pepck*, although Gliadin+ mice showed a lower ileal expression of *G6pase* (Supplementary Fig. S1). Furthermore, no effect on total liver weight was observed (Supplementary Fig. S1). Evaluation for non-alcoholic steatohepatitis parameters confirmed the higher steatosis grade of Gliadin+ mice, while all mice displayed the same degree of lobular inflammation and hepatocellular ballooning after 23 weeks of HFD (Supplementary Fig. S1). The dysregulated lipid phenotype of Gliadin+ mice did not manifest in the triglyceride profile (Supplementary Table S2) or in the expression levels of a series of lipid metabolism related genes in the liver, nor in the levels of alanine aminotransferase in plasma (Supplementary Fig. S1), a marker of liver damage. Likewise, the weight of the eWAT and expression levels of genes directly related to lipo- and adipogenic properties were unaffected (Supplementary Fig. S1). Finally, body weight development did not differ between Gliadin− and Gliadin+ mice during 23 weeks of HFD, and no differences in feed consumption or feed conversion ratio assessed by feed consumption per body weight gain were observed (Supplementary Fig. S1).

Taken together, these results suggested that gliadin intake in itself only moderately affected glucose and lipid metabolism. This should however be interpreted in the context that gliadin was able to add even to the severe detrimental effect on metabolic dysregulation caused by HFD consumption for 23 weeks.

### Gliadin Intake Altered Gut Microbial Composition and Activity

**Figure 2:**
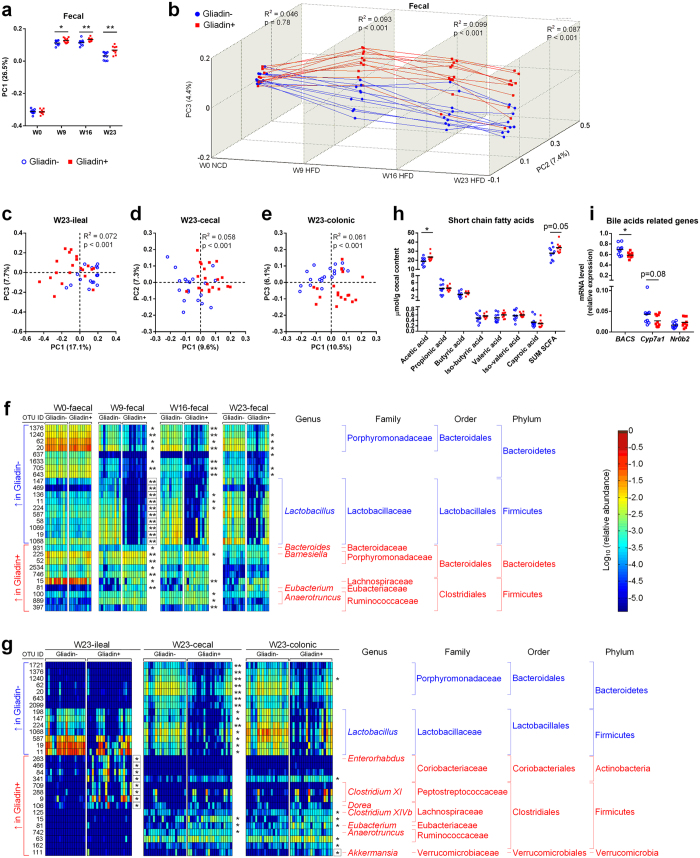
Gliadin intake altered intestinal microbial composition and activity. Microbiota analysis was performed on faecal samples collected from 10–11 cages per group at Week 0, 9, 16 and 23 and on terminal ileal, caecal and colonic luminal samples (n = 18–20 mice per group) by sequencing the V3 region of bacterial 16S rRNA genes followed by PCoA on unweighted UniFrac distances (**a**–**e**). Panel (a) shows that the HFD feeding, initiated after Week 0, affected the first coordinate (PC1) of the microbiota composition, while (b) shows the longitudinal effect of Gliadin feeding. Panels (c–e) show that Gliadin feeding affected the ileal, caecal and colonic microbiota in samples obtained at termination. In panel (a), asterisks represent significant differences between the two groups (*p < 0.05, **p < 0.01, unpaired t test or Mann-Whitney test), while in panels (b–e), p values are listed for differential clustering (ADONIS test) and R^2^ values represent the percentages of variation explained by gliadin intake. NCD designates Normal Chow Diet, HFD designates High-Fat Diet. Heatmaps (**f**,**g**) show the relative abundances of OTUs differing between the Gliadin− and Gliadin+ mice in faecal and intestinal samples. Taxonomy is reported at the lowest identifiable level. OTUs that are more abundant in the Gliadin+ group are indicated in red and those less abundant in blue. Statistical comparison of the two groups was done by 10,000 times of permutation; p values represent fraction of times that permuted differences assessed by Welch’s t test were greater than or equal to real differences, and were adjusted by FDR correction^71^ (*q < 0.05, **q < 0.01). Boxes surrounding asterisks indicate >ten-fold differences in OTU abundances between the two groups. For faecal samples, only OTUs that have at least one q < 0.02 are shown. Short chain fatty acid concentrations in caecum (**h**) and hepatic mRNA levels of bile acid related genes (i) were measured at termination (n = 9–10 non-fasted mice per group). The mean of each group is shown by a horizontal line. Asterisks represent statistically significant differences (*p < 0.05, unpaired t test or Mann-Whitney test). See also Supplementary Fig. S2.

To elucidate whether changes in the gut microbiome were accompanying the observed effects of gliadin on glucose and lipid metabolism, we sequenced the V3 region of the 16S rRNA genes in community DNA extracted from faecal samples at Weeks 0, 9, 16, and 23, and from terminal ileal, caecal, and colonic samples. As anticipated, gut microbiotas of both Gliadin− and Gliadin+ mice were strongly affected by HFD. Principal coordinate analysis (PCoA) clearly separated the Week 0 faecal microbiota from that obtained at Weeks 9, 16 and 23 (Fig. 2a), and alpha diversity was significantly reduced by the shift from normal chow diet at Week 0 to HFD in the following weeks (Supplementary Fig. S2).

PCoA analysis of unweighted UniFrac distance matrices furthermore showed a clear separation of the Gliadin− and Gliadin+ groups by their faecal microbiotas at Week 9, 16 and 23, as well as by ileal, caecal and colonic microbiotas at Week 23 (p values < 0.001, ADONIS test, Fig. 2b–e). The difference between the two groups was most significant in the ileal segments when assessed by weighted UniFrac distances (Supplementary Fig. S2). Interestingly, the ileal microbiota of Gliadin+ mice exhibited a significantly larger divergence than that of Gliadin− mice (within-group UniFrac distances: unweighted, 0.60 ± 0.07 versus 0.52 ± 0.09, weighted, 0.17 ± 0.08 versus 0.08 ± 0.08, p < 0.001), which is also manifested by the more scattered distribution of the Gliadin+ samples in the PCoA plots (Fig. 2c and Supplementary Fig. S2). This indicates individually divergent responses of the ileal microbiota to gliadin. No significant differences of α-diversity within faecal, ileal, caecal or colonic samples were observed when comparing the Gliadin− and Gliadin+ groups (Supplementary Fig. S2).

Gliadin intake altered the relative abundances of a total of 44 Operational Taxonomic Units (OTUs), of which the ones with more than ten-fold differences in relative abundance included OTUs identified as *Lactobacillus* that were less abundant in faecal samples of Gliadin+ mice at Week 9, as well as ileal OTUs identified as Coriobacteriaceae, *Enterorhabdus, Clostridium XI, Dorea*, and a colonic OTU identified as *Akkermansia* that were all more abundant in Gliadin+ mice (Fig. 2f and g). Findings at the genus/family level were generally consistent with the findings based on OTUs (Supplementary Fig. S2).

The observed effects of gliadin on microbial composition led us to examine whether also the gut microbial activity was affected. Total caecal short chain fatty acid (SCFA) concentrations tended to be higher in Gliadin+ mice than in Gliadin− mice (p = 0.05), which was mainly explained by higher levels of acetic acid (Fig. 2h). In feces, butyric acid was also higher in Gliadin+ mice than in Gliadin− mice (Supplementary Fig. S2). In relation to possible microbiome-induced effects on bile acid metabolism, we observed that the hepatic bile acyl-CoA synthetase gene (*Bacs*) was expressed at a significantly lower level in Gliadin+ mice (Fig. 2i), suggesting gliadin-induced changes in bile acid metabolism within the liver. Similarly, another gene regulated by the farnesoid X receptor, the cholesterol 7 alpha-hydroxylase gene (*Cyp7a*1), was expressed at a slightly (but not significantly) lower levels in Gliadin+ mice (p = 0.08, Fig. 2i).

### Gliadin Intake Caused Lower Expression of Gut Barrier Function Related Genes in Ileum

**Figure 3:**
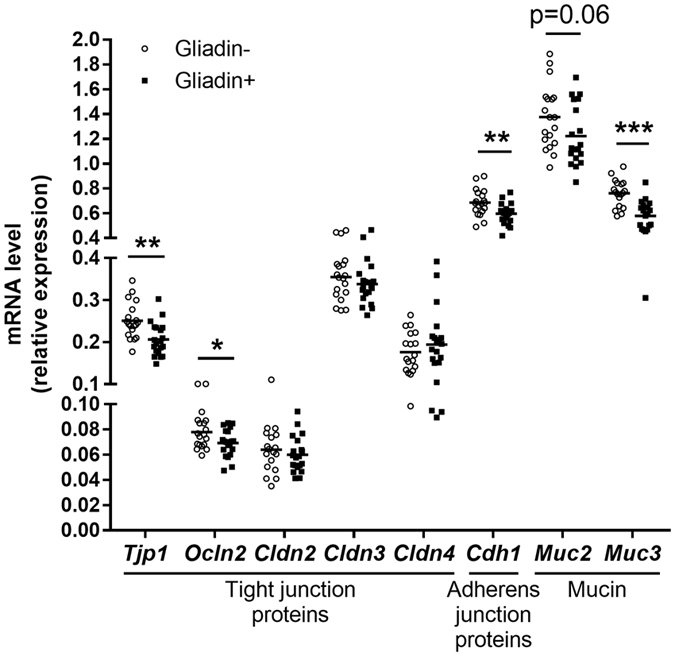
Gliadin intake decreased ileal expression of barrier function related genes. Ileal tissue mRNA levels of barrier function related genes in Gliadin− (n = 19) and Gliadin+ (n = 20) mice at termination. The mean of each group is shown by a horizontal line. Asterisks represent statistically significant differences between the two groups (*p < 0.05, **p < 0.01, ***p < 0.001, unpaired t test or Mann-Whitney test). See also Supplementary Fig. S3.

To analyse whether intake of gliadin affected the gut barrier function, we measured expression of genes in ileal and colonic tissues encoding five tight junction proteins including ZO-1 (*Tjp1*), occludin (*Ocln*), and claudin2-4 (*Cldn2, Cldn3, Cldn4*), as well as the adherens junction protein cadherin-1 (*Cdh1*) and two mucins (*Muc2, Muc3*). Gliadin+ mice showed lower ileal expression of *Tjp1, Ocln, Cdh1, Muc3*, and tended (p = 0.06) towards lower expression of *Muc2* (Fig. 3), indicating a disturbed barrier function. These effects were local to the ileum, as colonic expression of the same genes was similar between the two groups of mice (Supplementary Fig. S3), suggesting that gliadin mainly disturbs the ileal environment.

### Gliadin Intake Changed the Metabolic Signature of Urine

**Figure 4:**
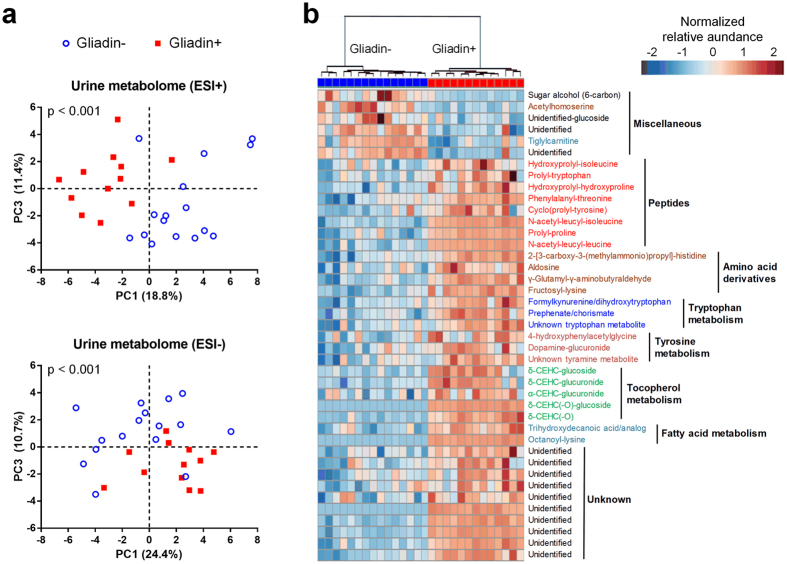
Gliadin affected the metabolic signature of urine. UPLC-MS-based global profiling of metabolites in urine from non-fasted Gliadin− (n = 15) and Gliadin+ (n = 13) mice at termination. PCA was performed on urine metabolome in positive (ESI+) and negative ionization (ESI−) mode respectively (**a**). Data were log-transformed and mean-centered. P values originate from Hotelling’s T^2^ test with 100,000 permutations. The heatmap (**b**) shows the normalized relative abundances of urinary metabolites differing between Gliadin− and Gliadin+ mice (q < 0.05, unpaired t test with Welch’s correction followed by FDR correction). Blue colors indicate relative abundances below and red indicate relative abundances above the mean of all samples. Data were log-transformed and the most abundant ion representing each metabolite was included. See also Supplementary Table S3.

Since intake of gliadin changed host physiology (Fig. 1), intestinal microbiota composition and activity (Fig. 2), along with gut barrier function (Fig. 3), we examined whether these changes were reflected in urine metabolite profiles. Exploratory metabolic profiling by Ultra Performance Liquid Chromatography Mass Spectrometry (UPLC-MS) of urine samples at Week 23 confirmed that intake of gliadin consistently modified the urinary metabolite signature. Principal component analysis (PCA) of metabolite profiles derived from both positive and negative ionization modes clearly separated Gliadin+ and Gliadin− mice (p values < 0.001, Hotelling’s T^2^ test, Fig. 4a).

In total we found 43 urinary metabolites to differ significantly in abundance between the two groups (Fig. 4b, Supplementary Table S3). Of these, the large majority (37 metabolites) were found in higher levels in Gliadin+ mice, indicating a higher intestinal permeability of Gliadin+ mice (Fig. 4b). Several of the metabolites were tentatively identified as intermediates and breakdown-products of amino acid metabolism, protein degradation, and tocopherol β-oxidation end products. Five dipeptides containing the amino acids proline or hydroxyproline were observed, most likely originating from gliadin itself. Furthermore, five oxidized tocopherol metabolites, carboxyethyl-hydroxychromans, were more abundant in Gliadin+ mice than in Gliadin− mice. Additionally, six metabolites associated with tyrosine and tryptophan metabolism including dopamine-glucuronide were identified among the metabolites more abundant in Gliadin+ mice than in Gliadin− mice, underpinning the observed gliadin-induced changes in the composition and/or activity of the gut microbiota, as these metabolites may be derived from intestinal bacteria.

### Gliadin Did Not Affect Systemic Inflammatory Markers but Altered Immune Cell Composition in Liver and Inflammatory Phenotype of Visceral Adipose Tissue

**Figure 5:**
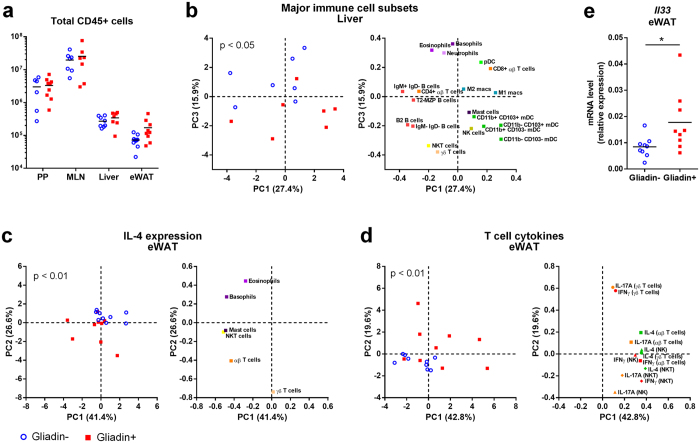
Gliadin altered the composition of immune cells in liver and inflammatory phenotype in eWAT. Composition of immune cells measured by flow cytometry of samples from Gliadin− and Gliadin+ non-fasted mice (n = 6–10 per group) at termination. Panel a shows total CD45+ leukocyte numbers in Peyer’s patches (PP), mesenteric lymph nodes (MLN), liver and eWAT. Horizontal lines represent means. Panel (b) shows a PCA of the major immune cell subsets in liver, (c) of the median fluorescence intensity of IL-4 in IL-4 expressing immune cells in eWAT, and (d) of the median fluorescence intensity of intracellular IFN-γ, IL-4 and IL-17A production in T and NK cells in eWAT. Panel (e) shows *Il33* mRNA levels in eWAT. In panels (a) and (e), the mean of each group is shown by a horizontal line, while asterisks represent statistically significant differences (*p < 0.05, unpaired t test or Mann-Whitney test). In panels (b–d), data were center log ratio-transformed and mean-centered, and p values originate from Hotelling’s T^2^ test with 100,000 permutations. See also Supplementary Figs S4–S6 and Supplementary Table S4.

Given the observed disturbance of gut barrier function and alterations in host and microbial metabolism, we speculated that gliadin might affect HFD-induced systemic inflammation. However, no gliadin-induced differences were found among the measured circulating cytokines (IL-1β, IL-6, IFNγ, TNFα and IL-10) at termination (Supplementary Fig. S4). Additionally, while the percentage of blood neutrophils was higher in Gliadin+ mice after 9 weeks, gliadin did not impact the percentage of blood monocytes and neutrophils after 23 weeks (Supplementary Fig. S4). Still, since multiple previous studies of metabolic dysregulation have emphasized the importance of specific immune cell subsets as a key etiological factor^27^,^28^, we examined whether immune responses within metabolic- and gut-related tissues were altered by gliadin intake. We performed a deep phenotyping of all major immune cell subsets within intestinal lymphoid tissues including Peyer’s patches and mesenteric lymph nodes, and within liver and eWAT (Supplementary Figs S4, S5, S6). Gliadin induced no statistically significant differences in the total number of leukocytes in any of the tissues (Fig. 5a). Notably, gliadin intake was not reflected in local differences in immune cell composition within intestinal Peyer’s patches and mesenteric lymph nodes (Supplementary Fig. S4), or in the phenotypes of these gut-associated immune cells (Supplementary Table S4).

Within the liver, we found that numbers of the major liver immune cell subsets were changed by gliadin as revealed in a PCA showing that Gliadin+ mice displayed a generally different immune profile mainly driven by higher numbers of innate-like cell types such as various myeloid dendritic cell subsets, NK, NKT, and γδ T cells (p < 0.05, Hotelling’s T^2^ test, Fig. 5b). Characterization of the inflammatory phenotype of the liver immune cell subsets through intracellular cytokine staining did not show any differences in immune cell activities between the two groups (Supplementary Table S4).

Within eWAT, the numbers of several of the immune cell subsets tended to be influenced by gliadin (Supplementary Fig. S4), but no general changes in the immune cell profiles were identified based on PCA analysis. However, a functional characterization of the activity of eWAT immune cell subsets revealed a gliadin-induced change in cellular levels of the anti-inflammatory IL-4 within several different immune cell subsets (Fig. 5c). IL-4 production was not different among eosinophils, generally considered as the primary immune cell type responsible for IL-4 secretion in adipose tissue^29^, but rather, IL-4 levels were higher in mast cells, the innate-like γδ T cells and NKT cells, as well as in antigen-specific αβ T cells within Gliadin+ mice (Fig. 5c and Supplementary Fig. S6). Additionally, we found that the overall levels of IL-4, IL-17A, and IFN-γ in both αβ T cells as well as in innate-like T cells and NK cells were higher in Gliadin+ mice (Fig. 5d), altogether showing that the overall activity of both adaptive and innate T cells, as well as NK cells in eWAT was altered by gliadin consumption. Moreover, higher gene expression levels of the IL-1 family cytokine IL-33, involved in adipose tissue homeostasis, were observed in Gliadin+ mice (Fig. 5e). Collectively, the data showed that gliadin intake modulated the inflammatory milieu in both eWAT and liver.

### Combining Alterations in Microbiome and Host Metabolic Features

**Figure 6:**
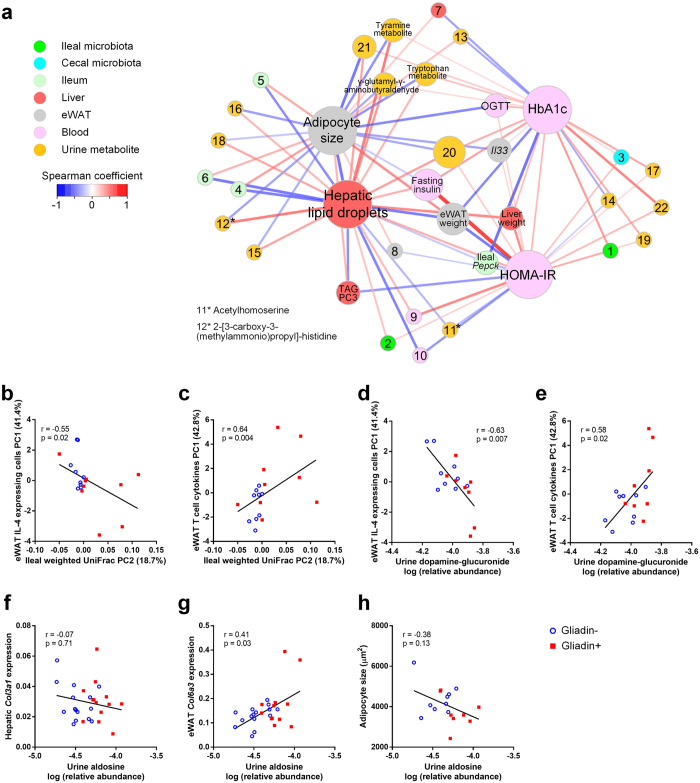
Combining Alterations in Microbiome and Host Metabolic Features. Panel (a) shows an interaction network built from Spearman correlations (p < 0.05) between four metabolic endpoints (hepatic lipid droplets, adipocyte size, HbA1c and HOMA-IR) and other parameters, including discriminating bacterial groups (q < 0.05) and microbial α diversity, discriminating urinary metabolites (q < 0.05), caecal SCFAs and other host parameters. Each node represents a parameter and the size of the node reflects the number of correlating nodes. Nodes associated with 3–4 key metabolic endpoints are shown by names instead of numbers. Lines represent correlations, and are colored red for positive and blue for negative correlations, while their thickness represents the strength of the correlation. Nodes are positioned using an organic layout in Cytoscape, and only nodes that connect to more than two other nodes are shown. 1, ileal *Clostridium XI*; 2, ileal *Enterorhabdus*; 3, caecal microbiota Shannon index; 4, ileal *Tjp1*; 5, ileal *Ocln*; 6, ileal *Cdh1*; 7, hepatic *Bacs*; 8, eWAT *Dgat1*; 9, circulating IL-1β; 10, circulating IFN-γ; 11*, acetylhomoserine; 12*, 2-[3-carboxy-3-(methylammonio)propyl]-histidine; 13, sugar alcohol (6-carbon); 14, unidentified glucoside; 15, N-acetyl-leucyl-isoleucine; 16, prolyl-proline; 17, trihydroxydecanoic acid/analog; 18, U148.1332; 19, U222.0441; 20, U301.212; 21, U344.2276; 22, U359.2176. Specific correlations (**b–h**) based on biological hypotheses generated by observations of Gliadin+ and Gliadin− samples. The individual hypotheses are described in the section ‘Combining Alterations in Microbiome and Host Metabolic Features’. P values and r coefficients of Spearman correlations are listed, and linear regression lines are shown. See also Supplementary Fig. S7.

Network analysis of significant Spearman correlations (p < 0.05) between the four gliadin-affected metabolic endpoints (i.e. hepatic lipid droplets, adipocyte size, HbA1c and HOMA-IR) and either microbiome-derived features (bacterial groups, diversity, SCFA), urinary metabolome, or other host parameters, respectively, revealed the most significantly interacting parameters with regard to the observed phenotypes (Fig. 6a). To narrow down correlations included in the network, which were not subjected to correction for multiple testing, only parameters correlating with at least two of the four metabolic endpoints were considered.

Fasting insulin, OGTT, eWAT weight, liver weight and triglyceride profile, did not (as described above) differ significantly between Gliadin− and Gliadin+ mice, but were correlated with three or all of the four differing metabolic endpoints, which supports the observed moderate effect of gliadin on metabolic dysregulation. Ileal gene expression of the rate-limiting gluconeogenesis gene *pepck* was negatively correlated with HbA1c, HOMA-IR and hepatic lipid droplets, which is in line with previous reports about the beneficial effects of intestinal gluconeogenesis on glucose and energy homeostasis. These include lowered food intake and lowered hepatic insulin sensitivity and consequently repression of hepatic gluconeogenesis via hepatoportal sensing of intestinal glucose levels^30^,^31^. Expression of *Il33* in eWAT also stood out in the network, negatively correlated with adipocyte size and positively correlated with HbA1c and HOMA-IR. Additionally, two groups of urinary metabolites altered by gliadin intake were of notice in the network. These two groups of gliadin-affected metabolites were associated with both the gut microbiota and the host metabolic parameters, and thus potentially contributed to the gliadin disturbed host-microbiota homeostasis. The first group comprises γ-glutamyl-γ-aminobutyraldehyde, and unknown metabolites of tryptophan and tyramine, which were all correlated to three of the gliadin-affected host parameters (Fig. 6a). These three metabolites are involved in the microbial metabolism of γ-aminobuturic acid (GABA), tryptophan and tyrosine^32^. GABA is a neurotransmitter, while tyrosine and tryptophan can be converted into the mood-determining molecules, dopamine and serotonin^32^, suggesting that gliadin intake may affect cognitive functions. The second group of urinary metabolites includes acetylhomoserine and 2-[3-carboxy-3-(methylammonio)propyl]-histidine. Acetylhomoserine, which was lower in the urine of Gliadin+ mice, is involved in bacterial synthesis of methionine^33^, while 2-[3-carboxy-3-(methylammonio)propyl]-histidine, higher in the urine of Gliadin+ mice, is the product of archaeal conversion of S-adenosyl methionine. Hepatic lipid droplet area was negatively correlated to the former but positively to the latter, which is in line with the fact that S-adenosyl methionine is depleted during chronic liver disease and is widely adopted as a therapy for the disease and intra-hepatic cholestasis^34^,^35^.

In addition to the associations related to metabolic endpoints differently affected in the two feeding groups, a large number of correlations were identified between the abundance of specific bacterial OTUs/phylotypes with different abundances in the two feeding groups, and given host parameters (Supplementary Fig. S7).

In our further exploration of correlated parameters, we focused on specific hypothesis-based correlations (Fig. 6b–h). In general, the Gliadin+ group displayed more variation within the ileal microbiota as well as in eWAT immune responses (Supplementary Fig. S2, Fig. 5c and d). We therefore hypothesized that the observed variations in gliadin-induced differences in immune responses in eWAT were caused by individual response patterns within the microbiota of the mice. This hypothesis was supported by correlations between the principal coordinate 2 (PC2) of the ileal microbiota profiles and principal component 1 (PC1) of eWAT IL-4 expressing cell types (Fig. 6b) and eWAT T-cell cytokine profiles (Fig. 6c), respectively. Strong correlations were also identified between urinary dopamine-glucuronide and immune response profiles in eWAT (Fig. 6d and e). This is in line with previous reports about interaction of dopamine with receptors in adipose tissues^36^,^37^. Finally, Gliadin+ mice displayed higher urinary levels of aldosine, which is an amino acid derived from aldol crosslinking of elastin and collagen. Urinary aldosine positively correlated with gene expression of *Col6a3* in eWAT, but not with *Col3a1* in the liver (Fig. 6f and g), suggesting that the higher aldosine levels were eWAT-derived. Increased collagen deposition in the adipose tissue would limit the capacity of adipocytes to expand^38^. However, no significant correlation between aldosine and adipocyte size was detected (Fig. 6h).

## Discussion

The impact of gluten in gluten-tolerant subjects is only scarcely elucidated although it may play an important role for development of life-style related diseases in populations with a high consumption of products based on refined wheat together with a high fat intake. Although a few studies have previously addressed effect of the combination of a HFD and gluten intake in mice^7^,^8^,^10^, conflicting results are reported, and the underlying mechanisms are not understood. We aimed to conduct a comprehensive explorative study that would take our current understanding of the interaction between gluten consumption and host metabolism a big step forward. We thus tested the effects of the specific wheat gluten component, gliadin, in a HFD mouse model, and found that a number of physiological parameters including long-term blood glucose levels (HbA1c), HOMA-IR, as well as total area of hepatic lipid droplets and eWAT adipocyte size, were affected by gliadin intake (Fig. 1). The use of completely defined diets allowed us to focus explicitly on the effect of a specific dietary component, and we find it notable that the relatively small change (exchanging casein for 4% gliadin) resulted in a considerable impact on the host response in the HFD mouse model.

**Figure 7:**
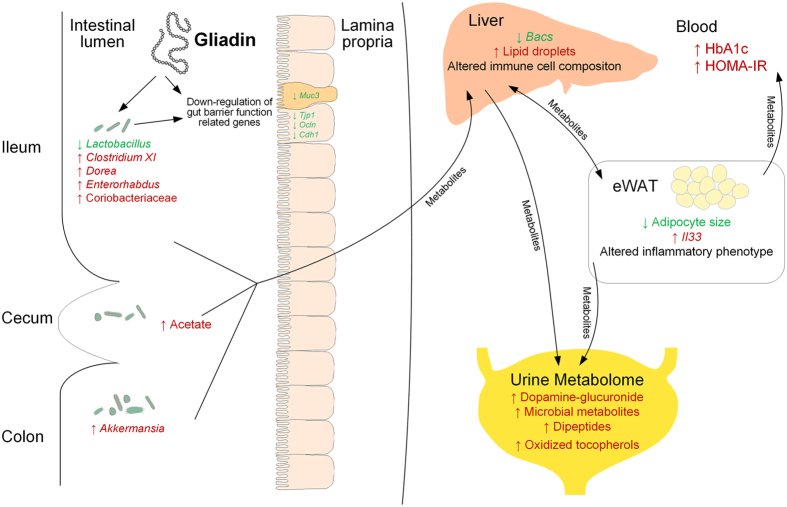
Schematic representation of the hypothesized effects of gliadin intake in a HFD-fed host. Ingested gliadin is not fully degraded by host digestive enzymes, leaving biologically active peptides in the gut. Gliadin peptides may directly affect gut barrier integrity, but can also alter the gut microbial composition and activities, thereby disturbing particularly the ileal gut barrier function. HFD together with increased gut permeability facilitate the influx of substances including microbial metabolites from the gut lumen to systemic circulation, affecting the metabolism and immune responses in extra-intestinal organs, including altered lipid metabolism and immune cell composition in the liver as well as altered inflammatory phenotype in the eWAT. The expandability of adipocytes in the eWAT is disturbed, resulting in reduced capacity for lipid storage and lipid spill-over to other organs, which subsequently causes increased hepatic lipid accumulation and increased systemic insulin resistance. Alterations in metabolism all over the body are reflected in the urine metabolite profile. Parameters higher in Gliadin+ mice are indicated in red, while parameters lower in Gliadin+ mice are indicated in green.

To elucidate the mechanisms behind the observed macroscopic effects, we investigated the impact of gliadin on gut microbiota, gut barrier function, urinary metabolome and immune responses in liver and adipose tissue (Fig. 7). Gliadin peptides are not fully degraded by host digestive enzymes^12^, and thus interact with both the host epithelium and the microbes present in the intestine. Accordingly, we observed a significant impact on the composition of the microbiota in the ileal and caecal environments, as well as on gut microbial activity, as reflected in a higher production of acetic acid and higher total SCFA levels (Fig. 2). As several previous reviews suggest that the gut microbiome affects intestinal integrity^39^,^40^, we speculate that not only the gliadin peptides themselves^14^,^15^, but also the gliadin-induced alterations in the ileal microbiota led to aberrations in the gut barrier function as measured by reduced expression of tight junction, adherens junction and mucin protein encoding genes (Fig. 3). Reduced intestinal barrier function in the gliadin-fed animals was additionally reflected in the observation that out of 43 specific urinary metabolites observed to be differentially abundant in gliadin-fed vs gliadin-free mice, 37 were found to be most abundant in the gliadin-fed mice (Fig. 4), indicating a generally increased leakiness of the gut mucosa. The mode of influx of bacterial components including lipopolysaccharides, or of dietary constituents, from the gut lumen to the systemic circulation, is thought to rely on passage through epithelial tight junctions or via chylomicron-facilitated transport from the gut^41^, two phenomena likely to be permitted by dietary intake of gliadin and HFD, respectively. In line with this, a recent study shows that dietary gluten can reach extra-intestinal organs including adipose tissues in HFD-fed mice^8^.

We suggest that the suspected increase in intestinal permeability caused by gliadin in the HFD mouse model led to translocation of dietary or bacterial components that affected the immune response in liver and adipose tissue as seen by a different immune cell profile with higher numbers of innate-like cell types in the liver (Figs. 5b and Supplementary Fig. S4), and a higher expression of the anti-inflammatory IL-4 by specific immune cells present in eWAT of gliadin-fed animals (Fig. 5c and Supplementary Fig. S6). Lipid-rich dendritic cells in the liver have been shown to be highly immunogenic, overexpress co-stimulatory molecules and CD1d, and are able to activate T cells, NKT cells and NK cells^42^. We speculate that increased flux of diet- or microbially derived lipid antigens to the liver might alter the liver immune cell composition via dendritic cell-mediated presentation of lipids and glycolipids to NK, NKT and γδ T cells. The IL-4-expressing immune cells were mainly of an innate-like T cell phenotype involving γδ T cells and NKT cells, but also included the memory-promoting αβ T cells (Fig. 5c). The gliadin-fed mice also showed higher *Il33* expression in the eWAT (Fig. 5e), which is in line with the idea that IL-33 favors an anti-inflammatory response^43^. However, adipose tissue T cells displayed an increased pro-inflammatory phenotype with higher levels of IFN-γ and IL-17A (Fig. 5d and Supplementary Fig. S6). This suggests the presence of several different types of antigens in eWAT. T cells react against different antigenic products (glycolipids, phospho- and peptide antigens), and some of the antigens could be constituted by peptides originating from gliadin breakdown, bacterial lipopolysaccharide, intact bacteria, or perhaps lipid products derived from rearrangement of the lipid pool within the adipose tissue. In line with this, translocation of intestinal bacteria to systemic circulation, mesenteric adipose tissue and mesenteric lymph nodes is known to increase with a high fat diet^44^, and a recent study of lard and fish oil based diets indicates that dietary lipid composition affects toll-like receptor-based activation within eWAT^45^. The heterogeneous inflammatory response in eWAT illustrates that multiple negative as well as counter-regulatory positive effects might be induced by gliadin. One example of heterogeneous inducers are diet-derived aryl hydrocarbon receptor ligands which have potent immune-modulatory characteristics and typically stimulate a Th17 response^46^, while they have also been shown to induce a Th1 response in adipocytes^47^. The extreme versatility of γδ and NK T cells upon ligand stimulation and potential influx to the adipose tissues of various heterogeneous substances may explain the mixed lymphocyte phenotypes^48^,^49^,^50^,^51^. Similarly, pro- as well as anti-inflammatory characteristics have previously been shown in local adipose tissue depots in Crohn’s Disease^52^.

The observed altered immune responses in eWAT may be related to the reduced size of adipocytes in the gliadin-fed animals. This is supported by the negative correlation between *Il33* expression and adipocyte cell size (Fig. 6a), which is in line with previous reports showing that IL-33 administration leads to reduced adipocyte size^53^, while IL-33 deficiency increases adipocyte size^43^. In a situation of energy surplus, limited adipose tissue expandability can be regarded as an adverse event, because an increased proportion of adipocytes with reduced capacity for lipid storage will lead to enhanced ectopic lipid deposition^54^, as reflected in more hepatic lipid accumulation in the gliadin-fed mice (Fig. 1c and d), and the strong negative correlation between adipocyte size and hepatic total lipid droplet area (Fig. 1h). Additionally, relative eWAT weight was negatively correlated to hepatic total lipid droplet area but positively to adipocyte size, further substantiating that adipose tissue dysregulation might have been causing the observed hepatic steatosis. In humans, a larger proportion of small adipocytes is found in insulin resistant than in insulin sensitive obese subjects^55^. Thus, the gliadin-disturbed adipocyte potential for lipid accumulation may contribute to systemic metabolic dysregulation, manifested as a gliadin-induced increase of HbA1c and HOMA-IR.

Analysis of the specific effects of bacterial group abundances within the intestinal microbial community (Fig. 2f and g) revealed that nine weeks of gliadin intake caused a more than ten-fold lower abundance of *Lactobacillus*, which is generally considered to be a beneficial member of the gut bacterial community. Furthermore, gliadin intake caused more than ten-fold higher abundances of *Clostridium* XI, *Dorea* and Coriobacteriaceae than seen in mice on a gliadin-free diet after 23 weeks. Strains belonging to *Clostridium* XI, including also the opportunistic pathogen *C. difficile*, are associated with compromised health^56^. *Dorea* spp. are found to be overrepresented in irritable bowel syndrome patients^57^,^58^, and patients with non-alcoholic fatty liver disease^59^. Coriobacteriaceae spp. have repeatedly been shown to be involved in host lipid metabolism^60^,^61^, and many bacteria within this group are considered as opportunistic pathogens^60^. Given these previously reported associations between diseases and *Clostridium* XI, *Dorea* and Coriobacteriaceae, which were all increased by the gliadin intake, we regard the observed changes to be adverse, and speculate that they might be involved in some of the detrimental metabolic responses identified after gliadin intake. In this regard it is also notable that *Akkermansia* was increased ten-fold in colonic samples from gliadin-fed animals (Fig. 2g). This genus has been linked to beneficial effects on metabolic health and inflammatory processes, and is suggested to be a biomarker for a healthy intestine^62^,^63^,^64^. *Akkermansia muciniphila* is specifically known for its ability to use mucins secreted from the epithelium as a sole carbon source^65^. As we observed lower expression of mucin genes in the ileum of gliadin-fed animals (Fig. 3) and a generally disturbed ileal environment, we speculate that a disturbed ileal mucosal turnover in these mice may have led to increased accessibility of mucins in colonic lumen, increasing the relative abundance of *Akkermansia* in this compartment (Fig. 2g). In addition, several microbial metabolites involved in neuronal signaling and S-adenosyl methionine metabolism were altered by gliadin intake and associated with metabolic phenotypes (Fig. 6a), and therefore may represent the mechanistic explanation for gliadin-disturbed host-microbiota homeostasis as described in the previous section.

The 4% gliadin content in feed adopted in this study was comparable to that in regular wheat flour (10–12% gluten, around 5% gliadin), while the fat content (35 g fat/100 g food) was high compared to that in an average human meal. Although our observations were done in mice and thus not necessarily translatable to humans, our findings suggest that the adverse effects of an obesogenic diet also in a human setting may be aggravated by consumption of gliadin-containing foods.

## Methods

### Animals

All animal experiments were approved by the Danish Animal Experiments Inspectorate and carried out in accordance with existing Danish guidelines for experimental animal welfare. Forty male C57BL/6NTac mice (Taconic, Lille Skensved, Denmark) aged four weeks at arrival were housed two by two and fed ad libitum with a standard rodent diet Altromin 1324 (Altromin, Lage, Germany) from Week 0. Experimental diets were fed starting from Week 1. For selected analyses, only one mouse from each cage was used. Details of the experiments are provided in the Supplementary Methods.

### Biochemical Measurements

Blood HbA1c, blood glucose, insulin, alanine aminotransferase and cytokines in plasma, as well as intestinal SCFAs were measured as described in the Supplementary Methods. Hepatic triglycerides were assessed by gas chromatography mass spectrometry as previously described^66^.

### Urine Metabolome Profiling

Urine samples were analysed by UPLC-MS as described in the Supplementary Methods.

### 16S rRNA Gene Sequencing and Analysis

Sequencing of the V3 region of bacterial 16S rRNA genes was performed with the Ion Torrent PGM system as previously described^67^. The average sequencing depth was 55,147 reads. Taxonomy was assigned with the Ribosomal Database Project Classifier v2.10.1^68^, and OTUs were generated with UPARSE v8.0.1623^69^, sorting 8,869,069 quality-filtered sequences into 2,932 OTUs at 97% sequence homology. Bioinformatical processing was performed with Qiime v1.8.0^70^. Further details are given in the Supplementary Methods. The microbial DNA sequences encoding bacterial 16S rRNA V3 regions reported in this paper have been deposited in the Sequence Read Archive (SRA) under the accession number SRP063048.

### Gene Expression Analysis by Real Time RT-PCR

Expression of gut barrier function related genes was determined by SYBR green based real time PCR (Supplementary Table S5), and the remaining analyses were based on Taqman primers and probes (Supplementary Table S6). Further details are given in the Supplementary Methods.

### Flow Cytometry

Anaesthetized mice were subject to intracardial perfusion with PBS followed by tissue harvest and preparation of single cell suspensions, which were analysed as described in the Supplementary Methods.

### Histology

Liver and adipose tissues were stained with Hematoxylin and Eosin stain, and subjected to histological analysis as described in the Supplementary Methods.

### Statistical Analysis

Unless specified, two-tailed Student’s t test (if normally distributed, evaluated by D’Agostino-Pearson test) or two-tailed Mann-Whitney test (if non-continuous data or not normally distributed) were performed using GraphPad Prism 6.02, and maximally one outlier from each group detected by Grubbs’ test (http://www.graphpad.com/quickcalcs/Grubbs1.cfm, alpha = 0.05) was excluded before these tests. Spearman’s rank correlation, multiple t tests and two-way ANOVA were also performed using GraphPad. Network based on Spearman correlations was built with Cytoscape v3.2.1.

## Additional Information

**How to cite this article**: Zhang, L. *et al*. Effects of Gliadin consumption on the Intestinal Microbiota and Metabolic Homeostasis in Mice Fed a High-fat Diet. *Sci. Rep.* **7**, 44613; doi: 10.1038/srep44613 (2017).

**Publisher's note:** Springer Nature remains neutral with regard to jurisdictional claims in published maps and institutional affiliations.

## Supplementary Material

Supplementary Information
