# Supplementary material for: Effects of Gliadin consumption on the Intestinal Microbiota and Metabolic Homeostasis in Mice Fed a High-fat Diet
Source: Sci Rep. 2017 Mar 16;7:44613. doi: 10.1038/srep44613 (PMC5353615; doi:10.1038/srep44613)
Supplement: Supplementary Information [file srep44613-s1.pdf]

SUPPLEMENTARY INFORMATION FOR:

Effects of Gliadin consumption on the Intestinal Microbiota and Metabolic Homeostasis in Mice Fed a High-fat Diet

Li Zhang, Daniel Andersen, Henrik Munch Roager, Martin Iain Bahl, Camilla Hartmann Friis Hansen, Niels Banhos Danneskiold-Samsøe, Karsten Kristiansen, Ilinca Daria Radulescu, Christian Sina, Henrik Lauritz Frandsen, Axel Kornerup Hansen, Susanne Brix, Lars I. Hellgren, Tine Rask Licht

CONTENT:

Supplementary Fig. S1. Additional host metabolic features and gene expression analyses.

Supplementary Fig. S2. Gliadin intake alters intestinal microbial composition and activity.

Supplementary Fig. S3. Gliadin intake did not affect colonic expression of barrier function related genes

Supplementary Fig. S4. Plasma cytokines and immune cell profiles.

Supplementary Fig. S5. Representative flow cytometry gating strategy

Supplementary Fig. S6. Representative intracellular cytokine staining of all CD3+ lymphocytes in eWAT.

Supplementary Fig. S7. Correlation heatmap.

Supplementary Table S1. Composition of the Gliadin- and Gliadin+ diets.

Supplementary Table S2. Hepatic triglyceride profile.

Supplementary Table S3. List of urinary metabolites differently abundant in Gliadin- and Gliadin+ mice.

Supplementary Table S4. Intracellular cytokines in immune cell subsets of Peyer's patches, mesenteric lymph nodes, liver and eWAT.

Supplementary Table S5. Primers used in SYBR green based real time PCR.

Supplementary Table S6. Primers and probes used in Taqman based real time PCR.

Supplementary Methods

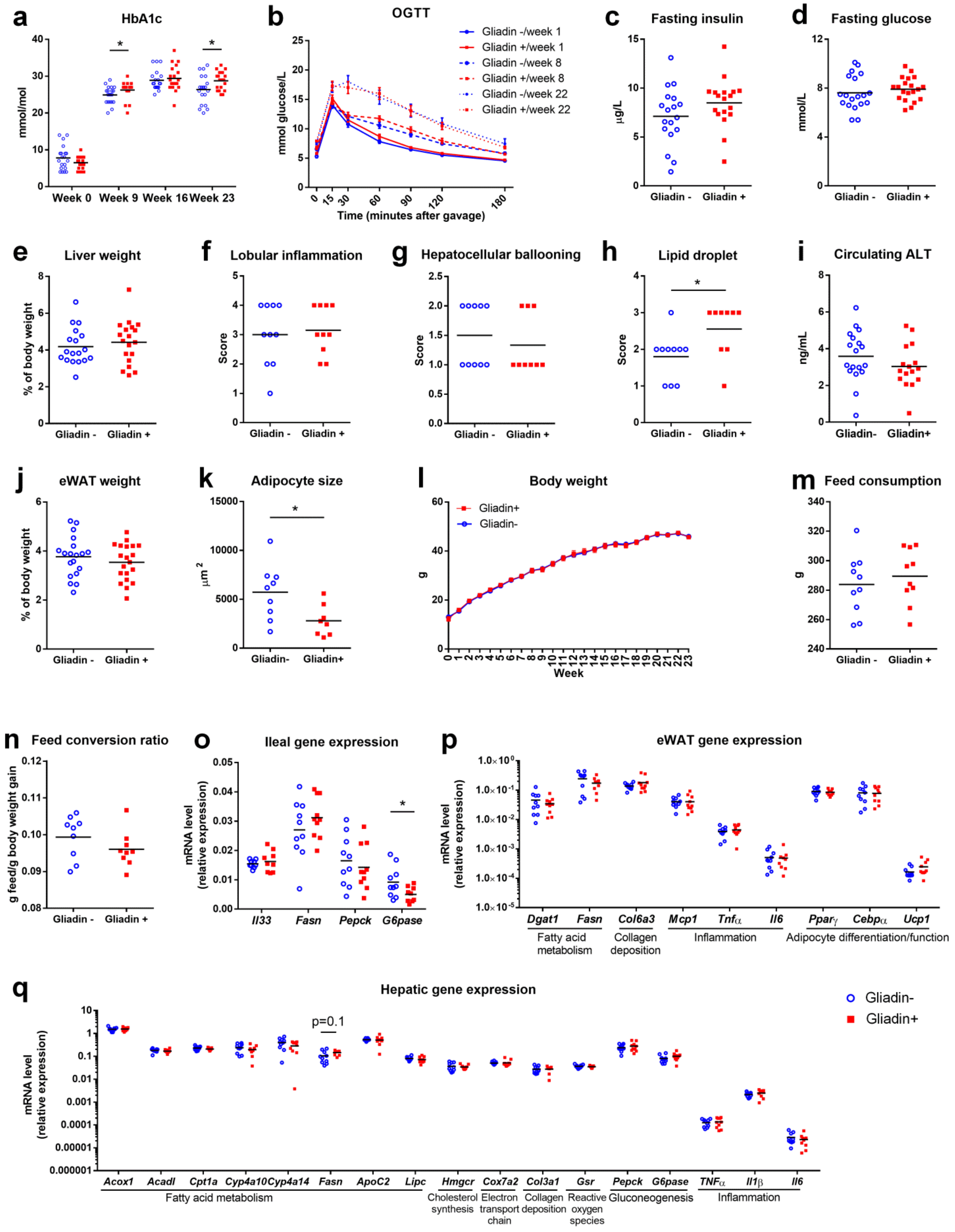
a
HbA1c
mmol/mol
Week 0
Week 9
Week 16
Week 23
b
OGTT
mmol glucose/L
Time (minutes after gavage)
Gliadin -/week 1
Gliadin +/week 1
Gliadin -/week 8
Gliadin +/week 8
Gliadin -/week 22
Gliadin +/week 22
c
Fasting insulin
µg/L
d
Fasting glucose
mmol/L
e
Liver weight
% of body weight
f
Lobular inflammation
Score
g
Hepatocellular ballooning
Score
h
Lipid droplet
Score
i
Circulating ALT
ng/mL
j
eWAT weight
% of body weight
k
Adipocyte size
µm 2
l
Body weight
g
Week
Gliadin+
Gliadin-
m
Feed consumption
g
n
Feed conversion ratio
g feed/g body weight gain
o
Ileal gene expression
mRNA level (relative expression)
Il33
Fasn
Pepck
G6pase
p
eWAT gene expression
mRNA level (relative expression)
Dgat1
Fasn
Col6a3
Mcp1
Tnfα
Il6
Pparγ
Cebpα
Ucp1
Fatty acid metabolism
Collagen deposition
Inflammation
Adipocyte differentiation/function
q
Hepatic gene expression
mRNA level (relative expression)
p=0.1
Acox1
Acadl
Cpt1a
Cyp4a10
Cyp4a14
Fasn
ApoC2
Lipc
Hmgcr
Cox7a2
Col3a1
Gsr
Pepck
G6pase
TNFα
Il1β
Il6
Fatty acid metabolism
Cholesterol synthesis
Electron transport chain
Collagen deposition
Reactive oxygen species
Gluconeogenesis
Inflammation
Gliadin-
Gliadin+
Gliadin -
Gliadin +

Supplementary Figure S1. Additional host metabolic features and gene expression analyses.

Panel a shows HbA1c levels in blood at Week 0, 9, 16 and 23, and Panel b shows OGTT at Week 1, 8 and 22. Panel c shows plasma levels of fasting insulin and d fasting glucose at Week 22. Panel e shows relative liver weight at Week 23, f-h show non-alcoholic steatohepatitis parameters including hepatic lobular inflammation, hepatocellular ballooning and lipid droplet size as evaluated by three independent pathologists, and i show plasma levels of alanine transaminase (ALT). Panel j shows relative eWAT weight at termination, and k shows eWAT adipocyte size measured using ImageJ software. Panel l shows body weight development during the 23 weeks of dietary intervention. Panels m-n show Cage-wise (n = 10-11 cages per group) measurements of total feed consumption per mouse and feed conversion ratio assessed by feed consumption per body weight gain. Panels o-q show mRNA levels of multiple genes in ileum, eWAT and liver. In panels a-e, i, j and l, n = 16-20 mice per group, while in panels f-h, k and o-q, n = 9-10 non-fasted mice per group. In panels a, c-k and m-q, the mean of each group is shown by a horizontal line. In panels b and l, data are shown as mean ± SEM. Asterisks represent significant differences between the two groups (*p < 0.05, unpaired t test or Mann-Whitney test).

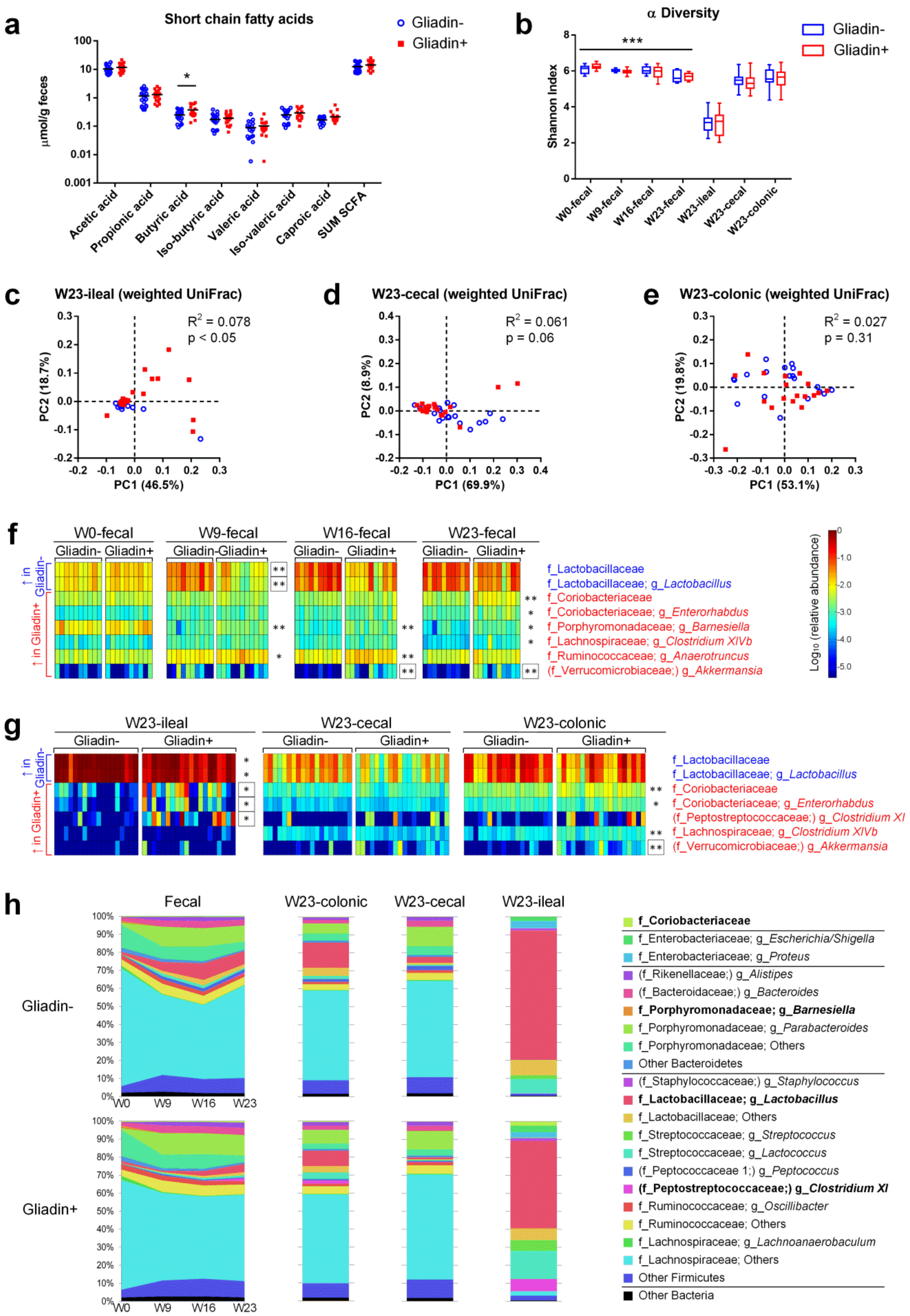
a
Short chain fatty acids
Gliadin-
Gliadin+
µmol/g feces
Acetic acid
Propionic acid
Butyric acid
Iso-butyric acid
Valeric acid
Iso-valeric acid
Caproic acid
SUM SCFA
b
α Diversity
Shannon Index
W0-fecal
W9-fecal
W16-fecal
W23-fecal
W23-ileal
W23-cecal
W23-colonic
c
W23-ileal (weighted UniFrac)
R2 = 0.078
p < 0.05
PC2 (18.7%)
PC1 (46.5%)
d
W23-cecal (weighted UniFrac)
R2 = 0.061
p = 0.06
PC2 (8.9%)
PC1 (69.9%)
e
W23-colonic (weighted UniFrac)
R2 = 0.027
p = 0.31
PC2 (19.8%)
PC1 (53.1%)
f
W0-fecal
W9-fecal
W16-fecal
W23-fecal
↑ in Gliadin-
↑ in Gliadin+
f_Lactobacillaceae
f_Lactobacillaceae; g_Lactobacillus
f_Coriobacteriaceae
f_Coriobacteriaceae; g_Enterorhabdus
f_Porphyromonadaceae; g_Barnesiella
f_Lachnospiraceae; g_Clostridium XIVb
f_Ruminococcaceae; g_Anaerotruncus
(f_Verrucomicrobiaceae;) g_Akkermansia
Log10 (relative abundance)
g
W23-ileal
W23-cecal
W23-colonic
f_Lactobacillaceae
f_Lactobacillaceae; g_Lactobacillus
f_Coriobacteriaceae
f_Coriobacteriaceae; g_Enterorhabdus
(f_Peptostreptococcaceae;) g_Clostridium XI
f_Lachnospiraceae; g_Clostridium XIVb
(f_Verrucomicrobiaceae;) g_Akkermansia
h
Fecal
W23-colonic
W23-cecal
W23-ileal
Gliadin-
Gliadin+
W0
W9
W16
W23
f_Coriobacteriaceae
f_Enterobacteriaceae; g_Escherichia/Shigella
f_Enterobacteriaceae; g_Proteus
(f_Rikenellaceae;) g_Alistipes
(f_Bacteroidaceae;) g_Bacteroides
f_Porphyromonadaceae; g_Barnesiella
f_Porphyromonadaceae; g_Parabacteroides
f_Porphyromonadaceae; Others
Other Bacteroidetes
(f_Staphylococcaceae;) g_Staphylococcus
f_Lactobacillaceae; g_Lactobacillus
f_Lactobacillaceae; Others
f_Streptococcaceae; g_Streptococcus
f_Streptococcaceae; g_Lactococcus
(f_Peptococcaceae 1;) g_Peptococcus
(f_Peptostreptococcaceae;) g_Clostridium XI
f_Ruminococcaceae; g_Oscillibacter
f_Ruminococcaceae; Others
f_Lachnospiraceae; g_Lachnoanaerobaculum
f_Lachnospiraceae; Others
Other Firmicutes
Other Bacteria

Supplementary Figure S2. Gliadin intake alters intestinal microbial composition and activity.
Panel a shows short chain fatty acid concentrations in feces of mice at termination (n = 19-20 mice per group). The mean of each group is shown by a horizontal line. Asterisks represent significant differences between the two groups (*p < 0.05, unpaired t test or Mann-Whitney test). Panel b shows microbial alpha diversity in feces (n = 10-11 cages per group) at Week 0, 9, 16 and 23, and in terminal samples from ileum, cecum and colon (n = 18-20 mice per group). Whiskers of the box plot represent the min and max values of each data set. Asterisks represent significant differences between fecal samples at Week 0, 9, 16 and 23 (***p < 0.001, two-way ANOVA).

Panels c-e show microbial beta diversity assessed by PCoA on weighted UniFrac distances between terminal samples from ileum, cecum and colon, respectively (n = 18-20 mice per group). P values are listed for differential clustering (ADONIS test), and R^2 values represent the percentage of variation explained by gliadin intake.
Panels f-g are heatmaps showing the relative abundances of bacterial groups differing between the Gliadin- and Gliadin+ mice. Bacteria that are more abundant in the Gliadin+ group are indicated in red and those less abundant in blue. Statistical comparison between the two groups was done by 10,000 times of permutation; p values represent fraction of times that permuted differences assessed by Welch's t test were greater than or equal to real differences, and were adjusted by FDR correction (*q < 0.05, **q < 0.01). Boxes surrounding asterisks indicate > ten-fold differences in abundances of bacterial groups between the two groups.
Panel h shows relative abundances of predominant bacterial groups. Groups that are differentially abundant between the Gliadin- and Gliadin+ mice are indicated in bold. In panels F-H, analyses were based on the phylotype data table. Taxonomies are reported at the lowest identifiable level, indicated by the letter preceding the underscore: f, family and g, genus. If one genus represents more than 90% of the family it belongs to, only the genus is shown, and the corresponding family is indicated with brackets.

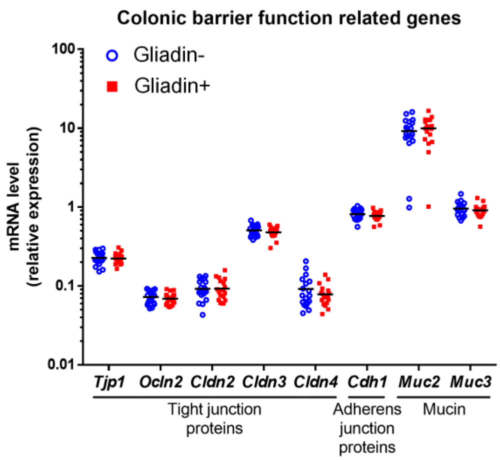

Supplementary Figure S3. Gliadin intake did not affect colonic expression of barrier function related genes.

The mean of each group (n = 19-20) is shown by a horizontal line. Asterisks indicate significant differences between the two groups (*p < 0.05, unpaired t test).

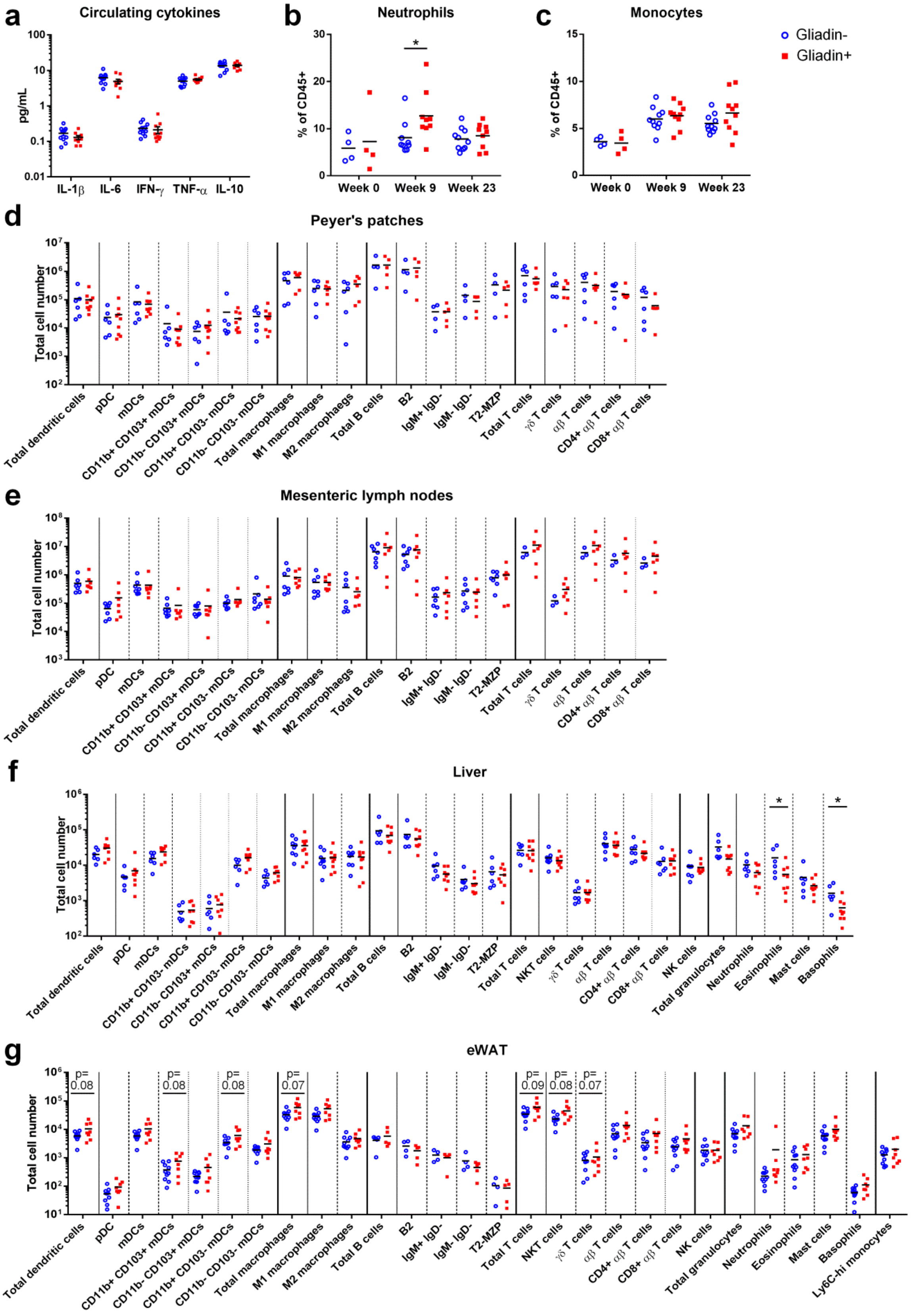
a
Circulating cytokines
pg/mL
IL-1β
IL-6
IFN-γ
TNF-α
IL-10
b
Neutrophils
% of CD45+
Week 0
Week 9
Week 23
c
Monocytes
% of CD45+
Gliadin-
Gliadin+
d
Peyer's patches
Total cell number
e
Mesenteric lymph nodes
f
Liver
g
eWAT
p=0.08
p=0.07
p=0.09
Total dendritic cells
pDC
mDCs
CD11b+ CD103+ mDCs
CD11b- CD103+ mDCs
CD11b+ CD103- mDCs
CD11b- CD103- mDCs
Total macrophages
M1 macrophages
M2 macrophages
Total B cells
B2
IgM+ IgD-
IgM- IgD-
T2-MZP
Total T cells
NKT cells
γδ T cells
αβ T cells
CD4+ αβ T cells
CD8+ αβ T cells
NK cells
Total granulocytes
Neutrophils
Eosinophils
Mast cells
Basophils
Ly6C-hi monocytes

Supplementary Figure S4. Plasma cytokines and immune cell profiles.

Panel a shows pro-inflammatory and anti-inflammatory cytokines in plasma measured by ELISA at Week 23 (n = 10 non-fasted mice). Panels b and c show frequencies of neutrophils and monocytes in peripheral blood (n = 4 pooled samples per group at Week 0, and n = 10 mice per group at Week 9 and 23). Panels d-g show total numbers of major immune cell subsets in Peyer's patches, mesenteric lymph nodes, liver and eWAT at Week 23 (n = 7-10 non-fasted mice per group). Asterisks represent significant differences between the two groups (*$p < 0.05$, unpaired t test or Mann-Whitney test).

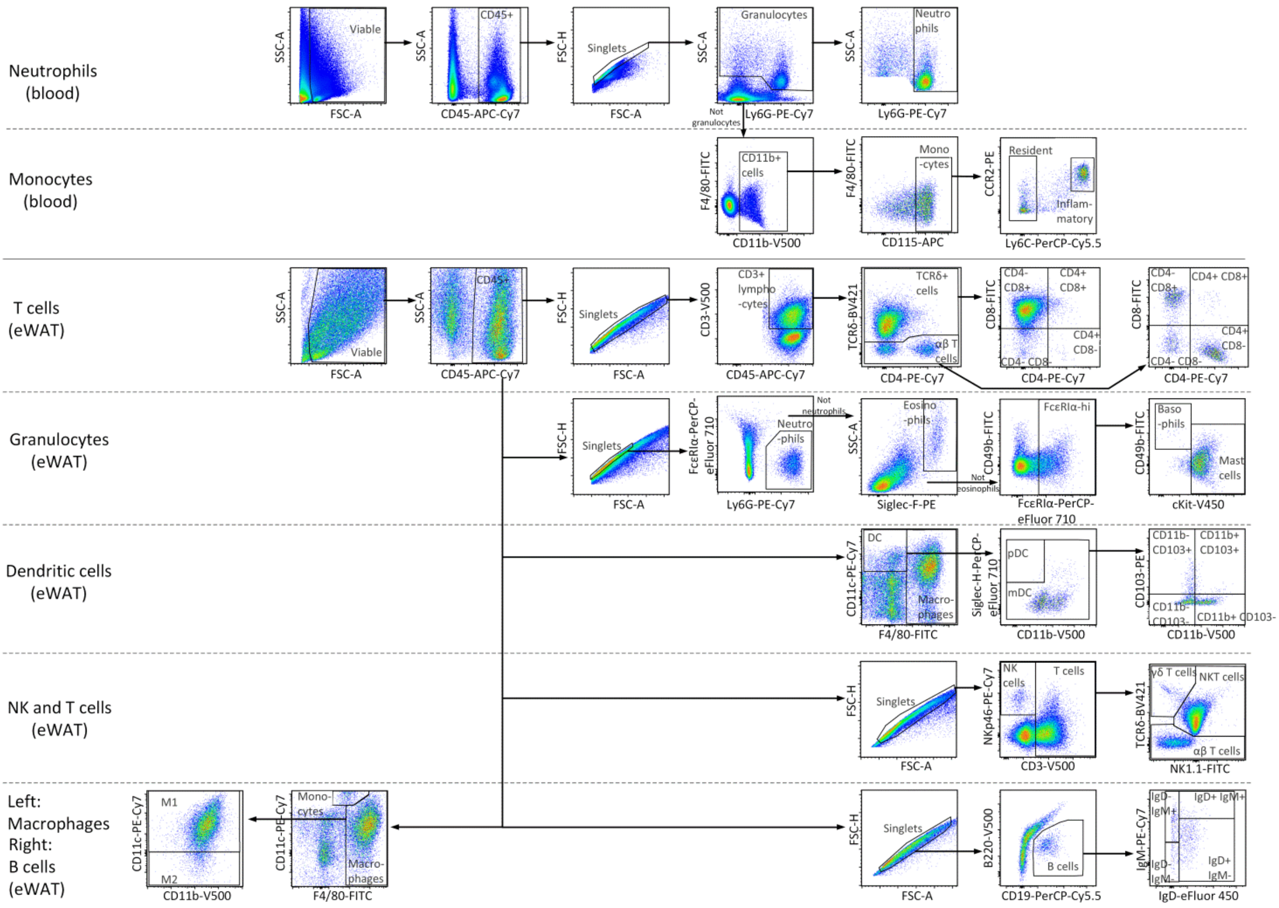

Supplementary Figure S5. Representative flow cytometry gating strategy.
Subsets of macrophages, dendritic cells, B cells, T cells, NK/NKT cells, and granulocytes in eWAT as well as neutrophils and monocytes in blood are shown.

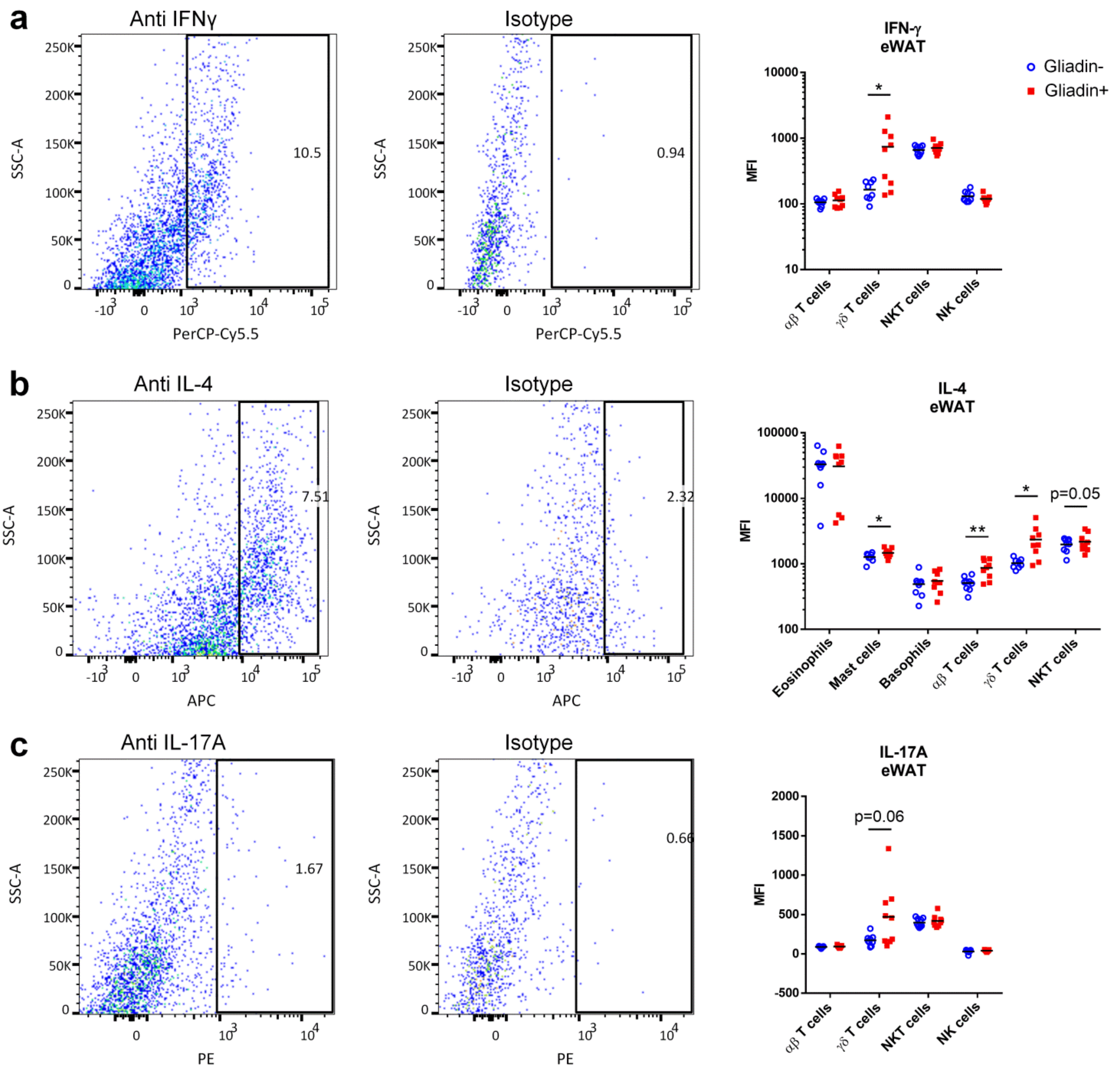

Supplementary Figure S6. Representative intracellular cytokine staining of all CD3+ lymphocytes in eWAT. IFN-γ, IL-4, IL-17A or appropriate isotype controls are shown.

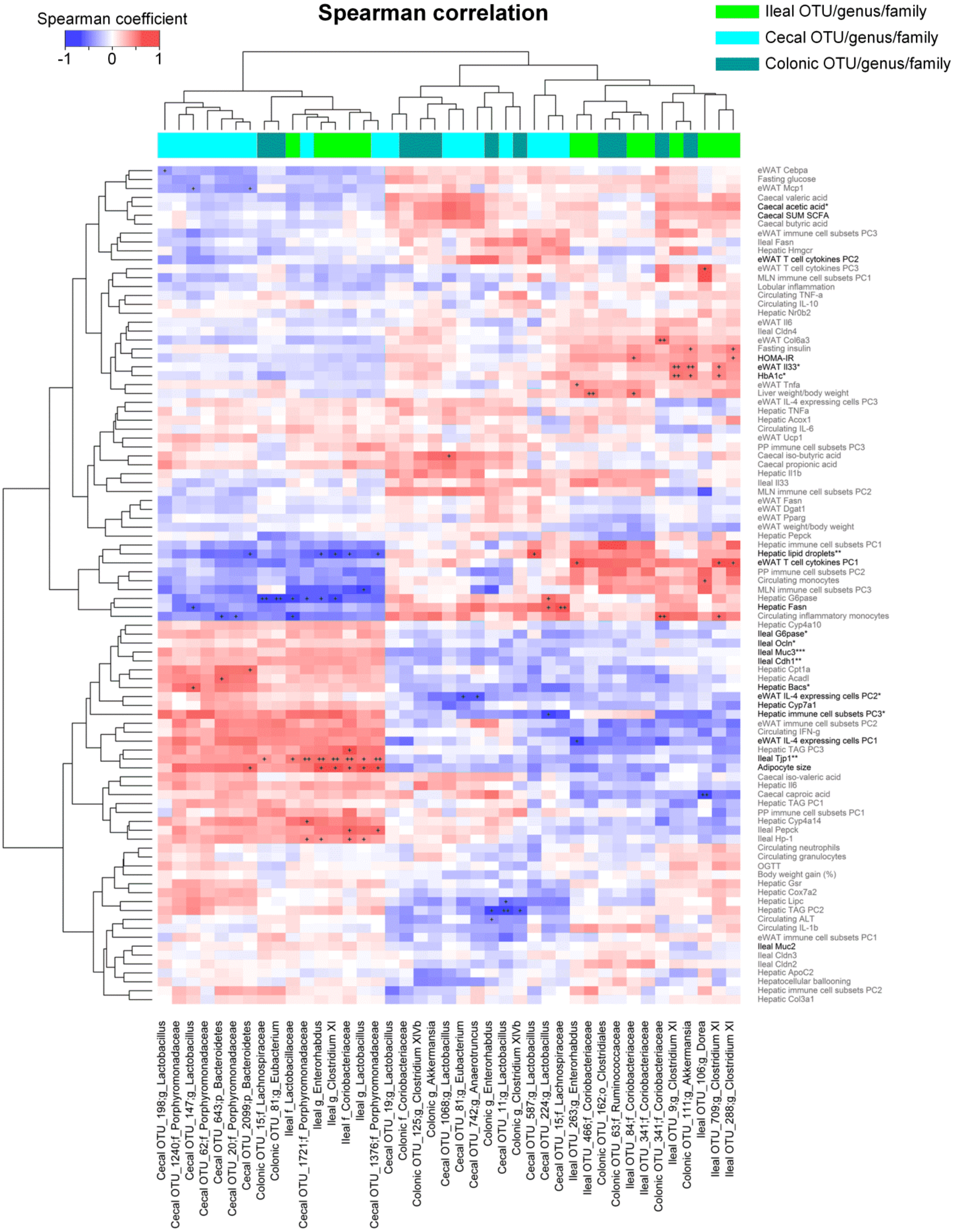

Spearman correlation
Spearman coefficient
-1
0
1
Ileal OTU/genus/family
Cecal OTU/genus/family
Colonic OTU/genus/family
eWAT Cebpa
Fasting glucose
eWAT Mcp1
Caecal valeric acid
Caecal acetic acid*
Caecal SUM SCFA
Caecal butyric acid
eWAT immune cell subsets PC3
Ileal Fasn
Hepatic Hmgcr
eWAT T cell cytokines PC2
eWAT T cell cytokines PC3
MLN immune cell subsets PC1
Lobular inflammation
Circulating TNF-a
Circulating IL-10
Hepatic Nr0b2
eWAT Il6
Ileal Cldn4
eWAT Col6a3
Fasting insulin
HOMA-IR
eWAT Il33*
HbA1c*
eWAT Tnfa
Liver weight/body weight
eWAT IL-4 expressing cells PC3
Hepatic TNFa
Hepatic Acox1
Circulating IL-6
eWAT Ucp1
PP immune cell subsets PC3
Caecal iso-butyric acid
Caecal propionic acid
Hepatic Il1b
Ileal Il33
MLN immune cell subsets PC2
eWAT Fasn
eWAT Dgat1
eWAT Pparg
eWAT weight/body weight
Hepatic Pepck
Hepatic immune cell subsets PC1
Hepatic lipid droplets**
eWAT T cell cytokines PC1
PP immune cell subsets PC2
Circulating monocytes
MLN immune cell subsets PC3
Hepatic G6pase
Hepatic Fasn
Circulating inflammatory monocytes
Hepatic Cyp4a10
Ileal G6pase*
Ileal Ocln*
Ileal Muc3***
Ileal Cdh1**
Hepatic Cpt1a
Hepatic Acadl
Hepatic Bacs*
eWAT IL-4 expressing cells PC2*
Hepatic Cyp7a1
Hepatic immune cell subsets PC3*
eWAT immune cell subsets PC2
Circulating IFN-g
eWAT IL-4 expressing cells PC1
Hepatic TAG PC3
Ileal Tjp1**
Adipocyte size
Caecal iso-valeric acid
Hepatic Il6
Caecal caproic acid
Hepatic TAG PC1
PP immune cell subsets PC1
Hepatic Cyp4a14
Ileal Pepck
Ileal Hp-1
Circulating neutrophils
Circulating granulocytes
OGTT
Body weight gain (%)
Hepatic Gsr
Hepatic Cox7a2
Hepatic Lipc
Hepatic TAG PC2
Circulating ALT
Circulating IL-1b
eWAT immune cell subsets PC1
Ileal Muc2
Ileal Cldn3
Ileal Cldn2
Hepatic ApoC2
Hepatocellular ballooning
Hepatic immune cell subsets PC2
Hepatic Col3a1
Cecal OTU_198;g_Lactobacillus
Cecal OTU_1240;f_Porphyromonadaceae
Cecal OTU_147;g_Lactobacillus
Cecal OTU_62;f_Porphyromonadaceae
Cecal OTU_643;p_Bacteroidetes
Cecal OTU_20;f_Porphyromonadaceae
Cecal OTU_2099;p_Bacteroidetes
Colonic OTU_15;f_Lachnospiraceae
Colonic OTU_81;g_Eubacterium
Ileal f_Lactobacillaceae
Cecal OTU_1721;f_Porphyromonadaceae
Ileal g_Enterorhabdus
Ileal g_Clostridium XI
Ileal f_Coriobacteriaceae
Ileal g_Lactobacillus
Cecal OTU_1376;f_Porphyromonadaceae
Cecal OTU_19;g_Lactobacillus
Colonic f_Coriobacteriaceae
Colonic OTU_125;g_Clostridium XIVb
Colonic g_Akkermansia
Cecal OTU_1068;g_Lactobacillus
Cecal OTU_81;g_Eubacterium
Cecal OTU_742;g_Anaerotruncus
Colonic g_Enterorhabdus
Cecal OTU_11;g_Lactobacillus
Colonic g_Clostridium XIVb
Cecal OTU_587;g_Lactobacillus
Cecal OTU_224;g_Lactobacillus
Cecal OTU_15;f_Lachnospiraceae
Ileal OTU_263;g_Enterorhabdus
Ileal OTU_466;f_Coriobacteriaceae
Colonic OTU_162;o_Clostridiales
Colonic OTU_63;f_Ruminococcaceae
Ileal OTU_84;f_Coriobacteriaceae
Ileal OTU_341;f_Coriobacteriaceae
Colonic OTU_341;f_Coriobacteriaceae
Ileal OTU_9;g_Clostridium XI
Colonic OTU_111;g_Akkermansia
Ileal OTU_106;g_Dorea
Ileal OTU_709;g_Clostridium XI
Ileal OTU_288;g_Clostridium XI

Supplementary Figure S7. Correlation heatmap.

Spearman correlations between host parameters/cecal SCFAs and OTUs/bacterial groups (genus/family level) that are differently abundant ($q < 0.05$) in Gliadin- and Gliadin+ mice. The letter preceding the underscore indicates the taxonomic level: f, family and g, genus. Plus signs represent correlation p values (+$p < 0.01$, ++$p < 0.001$). For row-names, parameters not differing between the two groups are indicated in gray; parameters with $p < 0.1$ are indicated in black; asterisks indicate significant differences between the two groups (*$p < 0.05$, **$p < 0.01$, unpaired t test or Mann-Whitney test). Clustering was performed using Euclidean distances of Spearman coefficients.

Supplementary Table S1. Composition of the Gliadin- and Gliadin+ diets.

		Gliadin-		Gliadin+	
		gm%	kcal%	gm%	kcal%
Protein		26.23	20.01	26.23	20.01
	Gliadin, Sigma G3375	0	0	4.01	3.06
	Casein	25.84	19.72	21.84	16.66
	L-Cystine	0.39	0.30	0.39	0.30
Carbohydrate		31.50	19.10	31.50	19.10
	Maltodextrin 10	16.15	12.32	16.15	12.32
	Sucrose	8.89	6.78	8.89	6.78
	Cellulose, BW200	6.46	0	6.46	0
Fat		34.89	59.90	34.89	59.90
	Soybean Oil	3.23	5.55	3.23	5.55
	Lard	31.66	54.35	31.66	54.35
Other		7.37	0.99	7.37	0.99
	Mineral Mix, S10026	1.29	0	1.29	0
	DiCalcium Phosphate	1.68	0	1.68	0
	Calcium Carbonate	0.71	0	0.71	0
	Potassium Citrate, 1 H_2O	2.13	0	2.13	0
	Vitamin Mix, V10001	1.29	0.99	1.29	0.99
	Choline Bitartrate	0.26	0	0.26	0
	FD&C Red Dye #40	0	0	0	0
	FD&C Blue Dye #1	0.01	0	0.01	0
Total		100		100	
kcal/gm		5.2		5.2	
Amino acid		(gm%)[a]		(gm%)[b]	
	Aspartate	0.91		0.78	
	Asparagine	1.04		0.98	
	Threonine	0.65		0.56	
	Serine	2.08		1.96	
	Glutamate	3.12		2.71	
	Glutamine	1.95		3.06	
	Proline	2.21		2.44	
	Glycine	1.17		1.09	
	Alanine	1.17		1.07	
	Valine	1.43		1.41	
	Methionine	0.65		0.59	
	Isoleucine	1.43		1.40	
	Leucine	2.21		2.21	
	Tyrosine	1.30		1.24	
	Phenylalanine	1.04		1.02	
	Lysine	1.82		1.55	
	Histidine	0.65		0.65	
	Tryptophan	0.26		0.28	
	Arginine	0.78		0.75	
	Cysteine	0.39		0.47	
	total	26.23		26.23	

[a]Calculation based on α-s1 casein; [b]Calculation based on α-gliadin.

Supplementary Table S2. Hepatic triglyceride profile.

	Gliadin-		Gliadin+		p value
	Concentration (mg/g)	Standard deviation	Concentration (mg/g)	Standard deviation	
Myristic acid (C14:0)	1.70	0.55	1.88	0.56	0.51
Pentadecyclic acid (C15:0)	1.02	0.56	1.10	0.64	0.80
Palmitic acid (C16:0)	88.10	25.25	94.03	27.83	0.65
Palmitoleic acid (C16:1)	6.63	2.48	7.85	2.80	0.36
Margaric acid (C17:0)	0.76	0.21	0.81	0.18	0.58
Stearic acid (C18:0)	5.22	1.41	6.08	1.40	0.22
Oleic acid (C18:1 n-9 cis)	114.29	31.93	133.37	41.88	0.31
Vaccenic acid (C18:1 n-7)	6.53	2.54	7.77	3.28	0.40
Linoleic acid (C18:2 n-6 cis)	93.97	30.89	90.89	29.39	0.84
γ-linoleic acid (C18:3 n-6)	3.18	1.42	2.96	1.15	0.73
Arachidic acid (C20:0)	0.63	0.27	0.72	0.27	0.52
α-linolenic acid (C18:3 n-3)	3.37	1.39	3.14	1.20	0.72
Gondoic acid (C20:1 n-9)	1.99	0.56	2.41	0.74	0.22
Dihomo-γ-linolenic acid (C20:3 n-6)	4.00	1.53	4.88	2.07	0.34
Arachidonic acid (C20:4 n-6)	10.83	4.99	11.31	5.07	0.85
Erucic acid (C22:1 n-9)	0.12	0.10	0.14	0.10	0.75
Eicosatetraenoic acid (C20:4 n-3)	0.38	0.17	0.45	0.21	0.51
Eicosapentaenoic acid (C20:5 n-3)	1.42	0.71	1.46	0.72	0.91
Adrenic acid (C22:4 n-6)	2.89	1.37	3.09	1.54	0.78
Docosapentaenoic acid (C22:5 n-6)	1.44	0.79	1.53	1.13	0.84
Docosapentaenoic acid (C22:5 n-3)	3.43	1.67	3.61	1.81	0.83
Docosahexaenoic acid (C22:6 n-3)	12.29	5.99	14.00	7.79	0.62
Total triglycerides	378.65	116.84	410.53	135.51	0.61

Supplementary Table S3. List of urinary metabolites discriminating between Gliadin- and Gliadin+ mice.

Metabolite	Type	Adduct	Retention time (min)	Experimental m/z	Experimental unconjugated[e] m/z	Authentic standard m/z	Database /theoretical m/z	Mass error (mDa)	Gliadin+ versus Gliadin- (Non-fasted)		
									Mean ratio	P value below	Q value below
**Amino acid metabolites**											
Fructosyl-lysine[b]	Maillard reaction product	$[M+H]^+$	0.6	309.1656			309.1656	0.0	2.2	0.001	0.001
		$[M+K]^+$	0.6	347.1217			347.1215	0.2	2.1	0.001	0.001
Dopamine glucuronide[a]	Dopamine metabolite	$[M+H]^+$	1.1	330.1181	154.0865	154.0865 Dopamine[a] $[M+H]^+$	330.1183	0.2	1.3	0.001	0.05
L-Formylkynurenine/ dihydroxytryptophan[b]	Tryptophan metabolite	$[M+H]^+$	1.4	237.0868			237.0870	0.2	1.8	0.001	0.05
2-[3-Carboxy-3-(methylammonio)propyl]-L-histidine[b]	Histidine metabolite	$[M+H]^+$	1.5	271.1399			271.1401	0.2	2.0	0.001	0.001
γ-Glutamyl-γ-aminobutyraldehyde	Microbial metabolite	$[M-H]^-$	2.7	215.1037			215.1026	1.1	3.2	0.001	0.001
Aldosine[b]	Amino acid derivate	$[M+H]^+$	3.1	255.1338			255.1339	0.1	1.8	0.001	0.01
Unknown tyramine metabolite[c] ($C_{11}H_{20}N_2O_4$)	Tyrosine metabolite	$[M+H]^+$	3.3	243.1335			243.1339	0.4	1.6	0.001	0.05
4-Hydroxyphenylacetylglycine[b]	Tyrosine metabolite	$[M+H]^+$	3.9	210.0759			210.0761	0.2	1.3	0.001	0.05
Prephenate/Chorismate[b]	Tyrosine/phenyl-alanine metabolite	$[M+H]^+$	4.0	227.0547			227.0550	0.3	1.9	0.001	0.001
Unknown tryptophan-metabolite[c] ($C_{13}H_{14}N_2O_4$)	Tryptophan-derived metabolite	$[M-H]^-$	4.2	261.0881			261.0870	1.1	1.7	0.001	0.001
		$[M+H]^+$	4.2	263.1026			263.1026	0.0	1.8	0.001	0.01
		$[M+Na]^+$	4.2	285.0844			285.0846	0.2	1.8	0.001	0.01
**Fatty acid related metabolites**											
Octanoyl-lysine[b]	Acyl carrier protein	$[M+H]^+$	4.5	273.2172			273.2173	0.1	169	0.001	0.001

	component									
Trihydroxydecanoic acid or analog[c]	Fatty acid	$[M-H]^-$	4.8	219.1237		219.1227	1.0	2.1	0.001	0.001
Tiglylcarnitine[b]	Acyl carnitine	$[M-H_2O-H]^-$	6.3	224.1289		224.1281	0.8	0.7	0.001	0.05
**Protein digestion products**										
Hydroxyprolyl-isoleucine[b]	Dipeptide	$[M+H]^+$	0.9	245.1495		245.1496	0.1	1.3	0.001	0.05
Phenylalanyl-threonine[b]	Dipeptide	$[M+H]^+$	1.9	267.1338		267.1339	0.1	2.2	0.001	0.001
Hydroxyprolyl-hydroxyproline[b]	Dipeptide	$[M-H_2O-H]^-$	3.1	225.0873		225.0875	0.2	1.5	0.001	0.001
		$[M-H_2O+H]^+$	3.1	227.1030		227.1032	0.2	1.6	0.001	0.001
		$[M-H_2O+H]^+$ isotope	3.1	228.1057		228.1062	0.5	1.5	0.001	0.001
Prolyl-proline[b]	Dipeptide	$[M+H]^+$	3.3	213.1229		213.1234	0.5	7.1	0.001	0.001
N-acetyl-leucyl-leucine[b]	Acetyl-dipeptide	$[M+H]^+$	3.6	287.1967		287.1965	0.0	10.7	0.001	0.001
Prolyl-tryptophan[b]	Dipeptide	$[M+H]^+$	4.4	302.1497		302.1499	0.2	1.3	0.01	0.05
Cyclo(prolyl-tyrosine)[b]	Cyclic-dipeptide	$[M+H-H_2O]^+$	4.6	243.1135		243.1134	0.1	1.4	0.001	0.05
N-acetyl-leucyl-isoleucine[b]	Acetyl-dipeptide	$[M-H]^-$	5.2	285.1819		285.1809	1.0	75	0.001	0.001
		$[M+H]^+$	5.2	287.1964		287.1965	0.0	21.8	0.001	0.001
		$[M+Na]^+$	5.2	309.1785		309.1785	0.0	20.0	0.001	0.001
**Tocopherol metabolites**										
δ-CEHC (-O)[b]	Oxidized tocopherol	$[M+NH_4]^+$	5.0	252.1593		252.1594	0.1	4.0	0.001	0.001
δ-CEHC-glucoside (-O)[b]	Oxidized tocopherol	$[M+NH_4]^+$	4.4	414.2121	252.1596 $[M+NH_4]^+$	414.2122	0.1	108	0.001	0.001
δ-CEHC-glucoside[b]	Oxidized tocopherol	$[M+NH_4]^+$	5.1	430.2071	251.1275 $[M+H]^+$	430.2072	0.1	2.5	0.001	0.01
δ-CEHC-glucuronide[b]	Oxidized tocopherol	$[M+NH_4]^+$	5.1	444.1863	251.1280 $[M+H]^+$	444.1864	0.1	2.7	0.001	0.01
α-CEHC-glucuronide[b]	Oxidized tocopherol	$[M-H]^-$	5.5	453.1770	277.1451 $[M-H]^-$	453.1755	0.5	1.4	0.001	0.05
	Oxidized tocopherol	$[M+NH_4]^+$	5.5	472.2176	279.1591 $[M+H]^+$	472.2177	0.1	1.4	0.01	0.05
**Other metabolites**										
Sugar alcohol (6-carbon) [c]	Sugar derivative	$[M-H]^-$	0.6	181.0718		181.0707	1.1	0.4	0.002	0.10

		$[M+H]^+$	0.6	183.0864		183.0863	0.1	0.3	0.001	0.01
Acetylhomoserine[b]	-	$[M-H]^-$	1.1	160.0616		160.0604	1.2	0.9	0.01	0.13
		$[M+H]^+$	1.1	162.0761		162.0761	0.0	0.7	0.001	0.01
**Unidentified metabolites[d]**						-				
Unidentified	-	$[M+NH_4]^+$	0.7	148.1332		-		2.9	0.001	0.001
Unidentified-Glucoside	-	$[M+H]^+$	0.7	441.1003	279.0477 $[M+H]^+$	-		0.4	0.001	0.05
Unidentified	-	$[M+H]^+$	0.8	184.0756		-		1.5	0.001	0.01
Unidentified (Not Tyramine-sulfate)	-	$[M-H]^-$	1.0	216.0344		-		259	0.001	0.001
Unidentified	-	$[M-H]^-$	1.1	222.0441		-		3.0	0.001	0.001
		$[M+H]^+$	1.1	224.0587		-		3.0	0.001	0.001
Unidentified	-	$[M+H]^+$	1.2	259.1651		-		0.4	0.001	0.001
Unidentified	-	$[M+H]^+$	4.1	277.1181		-		1.4	0.001	0.01
Unidentified	-	$[M+H]^+$	4.1	344.2276		-		1.4	0.01	0.05
Unidentified	-	$[M+H]^+$	4.4	301.2120		-		3.2	0.001	0.001
Unidentified	-	$[M+H]^+$	4.4	302.1497		-		1.4	0.01	0.05
Unidentified	-	$[M+H]^+$	4.6	359.2176		-		14.8	0.001	0.001
Unidentified	-	$[M+H]^+$	4.7	327.2024		-		38.0	0.001	0.001
Unidentified	-	$[M-H]^-$	5.3	189.1131		-		1.6	0.001	0.05
Unidentified	-	$[M+H]^+$	5.7	270.1699		-		0.8	0.001	0.01
Unidentified	-	$[M-H]^-$	5.8	286.1658		-		1.5	0.001	0.05
		$[M+H]^+$	5.8	288.1802		-		1.7	0.001	0.001
		$[M+Na]^+$	5.8	310.1624		-		2.0	0.001	0.01

[a]Identified using commercial standard. [b]Tentatively identified using MS/MS and METLIN and HMDB databases. [c]Putatively characterized compound class. [d]Unidentified and unclassified metabolite. [e]Unconjugated mass obtained measured by MS/MS experiment or glucuronidase treatment. CEHC, carboxyethyl-hydroxychroman.

Supplementary Table S4. Intracellular cytokines in immune cell subsets of Peyer's patches, mesenteric lymph nodes, liver and eWAT.

Cytokine	Population	Peyer's patches			Mesenteric lymph nodes			Liver			eWAT		
		Gliadin-	Gliadin+	P value	Gliadin-	Gliadin+	P value	Gliadin-	Gliadin+	P value	Gliadin-	Gliadin+	P value
IL-12p35	All DCs	1217.6 ± 260.4	1290.3 ± 221.5	0.58	1764.9 ± 299.9	1543.4 ± 221.0	0.17	1844.8 ± 644.7	2227.0 ± 1205.1	0.48	903 ± 111.5	1003 ± 155.4	0.18
	mDC	1304.6 ± 212.9	1366.3 ± 236.7	0.62	1745.4 ± 311.3	1580.9 ± 249.9	0.33	1956.3 ± 644.7	2265.9 ± 1254.5	0.58	900.4 ± 107.7	1003.5 ± 156.7	0.16
	pDC	979.1 ± 190.5	1001.7 ± 247.1	0.85	1651.7 ± 265.7	1318.7 ± 283.2	0.06	1243.9 ± 676.5	1467 ± 1166.2	0.68	904.5 ± 649.3	838.6 ± 531.0	0.83
	CD11b+ CD103+ mDC	1626.1 ± 248.7	1713.9 ± 533.7	0.69	2354.4 ± 504.4	2146.5 ± 365.2	0.43	2748 ± 1266.1	3655.1 ± 2387.1	0.40	1305.6 ± 285.3	1438.5 ± 205.5	0.30
	CD11b- CD103+ mDC	1232.7 ± 239.0	1171.9 ± 279.7	0.67	1695.9 ± 187.2	1458.1 ± 201.1	0.06	2018.8 ± 742.7	2460.9 ± 1205.9	0.43	994.9 ± 230.2	1010.9 ± 308.0	0.91
	CD11b+ CD103- mDC	1561.6 ± 309.6	1770.7 ± 425.7	0.31	1849.7 ± 458.8	1634.3 ± 309.4	0.36	1941.8 ± 613.6	2254.2 ± 1266.8	0.58	886.1 ± 115.4	976.6 ± 167.7	0.25
	CD11b- CD103- mDC	953.7 ± 160.6	1054.2 ± 188.0	0.30	1430.6 ± 189.0	1262.4 ± 171.6	0.13	1846.6 ± 655.2	2135.9 ± 1220.2	0.60	847.7 ± 77.1	953.1 ± 136.4	0.10
	Macrophages	1007 ± 139.6	961 ± 105.2	0.52	1310 ± 198.7	1119.1 ± 191.8	0.12	2049.7 ± 1076.1	2315.3 ± 1520.0	0.72	1039.2 ± 191.5	1289.3 ± 255.9	0.05
	M1 macrophages	965.9 ± 204.8	944.8 ± 113.3	0.83	1323 ± 237.1	1150.6 ± 189.9	0.19	2136.9 ± 1109.2	2381.3 ± 1582.6	0.75	1066.6 ± 196.2	1320.1 ± 264.8	0.05
	M2 macrophages	1054.7 ± 102.5	999.1 ± 148.5	0.42	1299.7 ± 214.7	1099.9 ± 189.8	0.11	1857.4 ± 1013.4	2185.8 ± 1403.0	0.63	893 ± 158.9	1114 ± 230.2	0.05
TNFa	Macrophages	2361.7 ± 633.2	1741.5 ± 694.8	0.17	9141.5 ± 2150.2	13955.8 ± 6050.8	0.11	2345.9 ± 1314.1	2177.6 ± 1341.6	0.79	1488.9 ± 283.5	1746.6 ± 655.5	0.33
	M1 macrophages	4494.3 ± 1408.8	3514.8 ± 1218.1	0.27	11104.5 ± 2398.9	15064.2 ± 7043.5	0.23	3026.7 ± 812.3	4199.5 ± 2828.3	0.26	1534.5 ± 283.7	1786.4 ± 665.7	0.34
	M2 macrophages	1015.7 ± 176.4	1023.3 ± 445.6	0.97	6442.6 ± 2583.6	9327.3 ± 4270.7	0.19	1629.7 ± 968.6	1128.8 ± 426.1	0.18	1271 ± 675.8	1572.4 ± 221.2	0.26
	Ly6C-hi monocytes	-	-	-	-	-	-	-	-	-	3204.5 ± 1238.5	2828.9 ± 580.5	0.45
IL-4	Neutrophils	NA	NA	NA	NA	NA	NA	11056 ± 3776.8	8832.2 ± 3870.5	0.27	6359.7 ± 1910.8	6815.1 ± 211.4	0.65
	Eosinophils	NA	NA	NA	NA	NA	NA	30507 ± 7340.7	29696.4 ± 12740.4	0.88	34862.7 ± 16606.9	30854.9 ± 19853.6	0.66

	Mast cells	NA	NA	NA	NA	NA	NA	5265.9 ± 3142.8	3471.3 ± 1821.3	0.21	1286.1 ± 167.0	1469.5 ± 208.4	<0.05
	Basophils	NA	NA	NA	NA	NA	NA	1615.5 ± 484.7	1300.6 ± 472.9	0.21	485.8 ± 166.3	548.8 ± 178.4	0.46
	T cells	NA	NA	NA	NA	NA	NA	1616.7 ± 256.2	1880.6 ± 1109.9	0.50	1654.6 ± 374.3	1853.7 ± 666.6	0.50
	αβ T cells	NA	NA	NA	NA	NA	NA	1309.4 ± 250.3	1549.5 ± 977.5	0.50	510.1 ± 112.0	868 ± 262.8	<0.01
	γδ T cells	NA	NA	NA	NA	NA	NA	1673.4 ± 346.4	2159.6 ± 1479.1	0.36	1120.8 ± 314.8	2352.6 ± 1217.9	<0.05
	NKT cells	NA	NA	NA	NA	NA	NA	3736 ± 836.4	4357 ± 2380.4	0.48	1980.1 ± 418.1	2176 ± 653.9	0.49
	NK cells	NA	NA	NA	NA	NA	NA	1109.9 ± 214.7	1272.6 ± 733.1	0.54	662.4 ± 347.3	1103.2 ± 483.1	0.05
IFN-γ	T cells	667.0 ± 674.1	506.4 ± 501.3		216 ± 41.0	238.6 ± 67.5		187.7 ± 30.0	174.7 ± 38.8	0.45	531.7 ± 87.4	542.9 ± 140.4	0.85
	αβ T cells	252.3 ± 51.1	305.4 ± 160.7	0.46	222.8 ± 48.0	234.1 ± 53.3	0.76	81 ± 10.0	93 ± 26.8	0.24	105.7 ± 11.9	113 ± 23.7	0.45
	γδ T cells	817.7 ± 981.5	469.2 ± 525.1	0.43	207.8 ± 79.9	821.4 ± 935.3	0.16	146.6 ± 29.4	180.4 ± 44.3	0.08	261.7 ± 277.8	740.8 ± 624.0	<0.05
	NKT cells	NA	NA	NA	NA	NA	NA	841.6 ± 172.9	780.8 ± 169.2	0.48	664.1 ± 91.7	710.3 ± 122.9	0.41
	NK cells	NA	NA	NA	NA	NA	NA	73.9 ± 10.3	84.3 ± 21.7	0.22	119.2 ± 15.6	130.9 ± 23.0	0.25
IL-17A	T cells	NA	NA	NA	NA	NA	NA	62.9 ± 10.5	53.3 ± 4.5	<0.05	310.7 ± 44.7	309.4 ± 49.4	0.96
	αβ T cells	NA	NA	NA	NA	NA	NA	31.7 ± 3.9	30.6 ± 6.0	0.65	87.8 ± 8.9	93.4 ± 15.3	0.39
	γδ T cells	NA	NA	NA	NA	NA	NA	52.8 ± 6.0	66.3 ± 17.7	0.05	173.8 ± 66.1	470.9 ± 371.4	0.06
	NKT cells	NA	NA	NA	NA	NA	NA	238.6 ± 36.4	208.3 ± 36.0	0.10	397.3 ± 52.2	417.9 ± 68.2	0.51
	NK cells	NA	NA	NA	NA	NA	NA	24.4 ± 6.5	30.3 ± 14.6	0.30	40.9 ± 10.4	30.5 ± 19.4	0.21

Data are shown as mean of the median fluorescence intensity ± standard deviation. mDC, myeloid dendritic cells, and pDC, plasmacytoid dendritic cells.

Supplementary Table S5. Primers used in SYBR green based real time PCR.

Gene	Primer	Reference
Pgk1	Forward CTCCGCTTTCATGTAGAGGAAG<br>Reverse GACATCTCCTAGTTTGGACAGTG	1
Gapdh	Forward CGACTTCAACAGCAACTCCCACTCTTCC<br>Reverse TGGGTGGTCCAGGGTTTCTTACTCCTT	2
Actb	Forward GTCCACCTTCCAGCAGATGT<br>Reverse GAAAGGGTGTAAAACGCAGC	3
Cldn2	Forward GTGGCTGTAGTGGGTGGAGT<br>Reverse CCAAAGAAAACAGGGCTGAG	4
Cldn3	Forward CCACTACCAGCAGTCGATGA<br>Reverse CAGCCTGTCTGTCCTCTTCC	5
Cldn4	Forward TCGCGCTTGGTAGCTGGTGC<br>Reverse GATCCCCAGCCAGCCCAGGA	6
Muc2	Forward GTGTGGGACCTGACAATGTG<br>Reverse ACAACGAGGTAGGTGCCATC	7
Muc3	Forward CGTGGTCAACTGCGAGAATGG<br>Reverse CGGCTCTATCTCTACGCTCTCC	8
Ocln	Forward ACTGGGTCAGGGAATATCCA<br>Reverse TCAGCAGCAGCCATGTACTC	9
Tjp1	Forward TGGGAACAGCACACAGTGAC<br>Reverse GCTGGCCCTCCTTTTAACAC	10
Cdh1	Forward GTATCGGATTTGGAGGGACA<br>Reverse CAGGACCAGGAGAAGAGTGC	11

Supplementary Table S6. Primers and probes used in Taqman based real time PCR.

Gene	Primer	Probe
Acadl	Forward AATATCTGAGTGGAGGCTGAAG<br>Reverse GCATCAACATCGCAGAGAAAC	/56-FAM/TACTTGGGA/ZEN/AGAGCAAGCGTACTCC/3IABkFQ/
Acox1	Forward TCATTCAAGTACGACACCATACC<br>Reverse ACACTGTTATGATGCTGCAGA	/56-FAM/TCCCCGACT/ZEN/GAACCTGGTCATAGA/3IABkFQ/
Apoc2	Forward AGGAGAGTAAGGAGCTGGTC<br>Reverse GAAGACATACCCGATCAGCAT	/56-FAM/CCATGAGCA/ZEN/CTTACGCAGGCATTT/3IABkFQ/
B2m	Forward GGGTGGAACTGTGTTACGTAG<br>Reverse TGGTCTTTCTGGTGCTTGTC	/56-FAM/CCGGAGAAT/ZEN/GGGAAGCCGAACATAC/3IABkFQ/
Ccl2	Forward AACTACAGCTTCTTTGGGACA<br>Reverse CATCCACGTGTTGGCTCA	/56-FAM/ACTCACCTG/ZEN/CTGCTACTCATTCACC/3IABkFQ/
Cebpa	Forward TCATTGTCACTGGTCAACTCC<br>Reverse ACAAGAACAGCAACGAGTACC	/56-FAM/CGCAAGAGC/ZEN/CGAGATAAAGCCAAAC/3IABkFQ/
Col3a1	Forward TCTCTAGACTCATAGGACTGACC<br>Reverse TTCTTCTCACCCTTCTTCATCC	/56-FAM/CATCTACGT/ZEN/TGGACTGCTGTGCCA/3IABkFQ/
Col6a3	Forward CATGTCTCCATCTGCTCCATC<br>Reverse ACGCTGAAGTTGTACCAGAAC	/56-FAM/TGAATGACT/ZEN/ACCTTCACAGTATCAGGCGA/3IABkFQ
Cox7a2	Forward GCATCCCATTATCCTCCTGAA<br>Reverse AGCCAAGATGTTGCGGAAT	/56-FAM/CGTGAAGTG/ZEN/GTGCTGATGGTCCT/3IABkFQ/
Cpt1a	Forward AGTGTCCATCCTCTGAGTAGC<br>Reverse CAGCAAGATAGGCATAAACGC	/56-FAM/ATGACATAC/ZEN/TCCCACAGATGGCCC/3IABkFQ/
Cyp4a10	Forward TCCATTCAACAAGAGCAAACC<br>Reverse TTCTGGGGAAGCAAGGC	/56-FAM/TTAGCCTTT/ZEN/GGATCTGATCGCCCC/3IABkFQ/
Cyp4a14	Forward CCACCTTCAGCTCGTTCATAG<br>Reverse CCAGATTCTTCTCACCATAGCC	/56-FAM/TCCCAATGC/ZEN/AGTTCCTTGATCCTCC/3IABkFQ/
Cyp7a1	Forward GTGAAGTCCTCCTTAGCTGTC<br>Reverse GCCATTTACTTGGATCAAGAGC	/56-FAM/CCGCAGAGC/ZEN/CTCCTTGATGATGC/3IABkFQ/
Dgat1	Forward CACCAGGATGCCATACTTGA<br>Reverse TCTTTGTTCAGCTCAGACAGTG	/56-FAM/AGCATCACC/ZEN/ACACACCAATTCAGGA/3IABkFQ/
Fasn	Forward ACTCCTGTAGGTTCTCTGACTC<br>Reverse GCTCCTCGCTTGTCGTC	/56-FAM/TGGCTCTTC/ZEN/TCTGTCTGGGCTCT/3IABkFQ/
G6pase	Forward GGAGGCTGGCATTGTAGATG<br>Reverse TCTACCTTGCTGCTCACTTTC	/56-FAM/TGGAGTCTT/ZEN/GTCAGGCATTGCTGT/3IABkFQ/
Gapdh	Forward AATGGTGAAGGTCGGTGTG<br>Reverse GTGGAGTCATACTGGAACATGTAG	/56-FAM/TGCAAATGG/ZEN/CAGCCCTGGTG/3IABkFQ/
Gsr	Forward AGCATCTCATCACAGCCAATC<br>Reverse TCTACTCGACTGCCTTTACCC	/56-FAM/TGCCAACCA/ZEN/CCTTTTCCTCTTTGTTG/3IABkFQ/
Hmgcr	Forward ACTGACATGCAGCCGAAG<br>Reverse CACATTCACTCTTGACGCTCT	/56-FAM/CATGGTGCC/ZEN/AACTCCAATCACAAGG/3IABkFQ/
Il1b	Forward CTCTTGTTGATGTGCTGCTG<br>Reverse GACCTGTTCTTTGAAGTTGACG	/56-FAM/TTCCAAACC/ZEN/TTTGACCTGGGCTGT/3IABkFQ/
Il33	Forward TCATGTTCACCATCAGCTTCT<br>Reverse GTGCTACTACGCTACTATGAGTC	/56-FAM/ACCGTCGCC/ZEN/TGATTGACTTGCA/3IABkFQ/
Il6	Forward TCCTTAGCCACTCCTTCTGT<br>Reverse AGCCAGAGTCCTTCAGAGA	/56-FAM/CCTACCCCA/ZEN/ATTTCCAATGCTCTCCT/3IABkFQ/
Lipc	Forward CCACTAACCCCACATTCACA<br>Reverse AGCCGCTTATCATGATCATCC	/56-FAM/AGCAAGCCA/ZEN/TCCACCGACCA/3IABkFQ/
Nr0b2	Forward TCCAAGACTTCACACAGTGC	/56-FAM/ATCCTCTTC/ZEN/AACCCAGATGTGCCAG/3IABkFQ/

	Reverse CAAGGAGTATGCGTACCTGAAG	
Pepck	Forward GCGAGTCTGTCAGTTCAATACC<br>Reverse GGATGTCGGAAGAGGACTTTG	/56-FAM/CATACATGG/ZEN/TGCGGCCTTTCATGC/3IABkFQ/
Pparg	Forward TGCAGGTTCTACTTTGATCGC<br>Reverse CTGCTCCACACTATGAAGACAT	/56-FAM/AGCTGACCC/ZEN/AATGGTTGCTGATTACA/3IABkFQ/
Bacs	Forward ACTCTCTCCACACATCTCCTC<br>Reverse ACGGTCATTCAGTACATTGGT	/56-FAM/CGGTCATTT/ZEN/GGTTTCTGCGGTGT/3IABkFQ/
Tnfa	Forward TCTTTGAGATCCATGCCGTTG<br>Reverse AGACCCTCACACTCAGATCA	/56-FAM/CCACGTCGT/ZEN/AGCAAACCACCAAGT/3IABkFQ/
Ucp1	Forward CACACCTCCAGTCATTAAGCC<br>Reverse CAAATCAGCTTTGCCTCACTC	/56-FAM/AAACACCTG/ZEN/CCTCTCTCGGAAACAA/3IABkFQ/

SUPPLEMENTARY METHODS

General materials

All aqueous solutions were prepared using ultrapure water obtained from the Milli-Q system (Merck Millipore, Bedford, MA, USA).

Animal experiments

Forty male C57BL/6NTac mice (Taconic, Lille Skensved, Denmark) aged four weeks at arrival were housed two by two and fed ad libitum a standard rodent diet Altromin 1324 (Altromin, Lage, Germany) at Week 0. From Week 1, one group of mice (n = 20) were fed a synthetic D12492 high-fat diet (Gliadin-) containing 60% of the energy from fat (54.4% of lard and 5.6% of soybean oil), while the other group of mice (n = 20) were fed a modified high-fat diet containing 4% gliadin (Gliadin+), replacing a corresponding amount of casein (Research Diets, New Brunswick, NJ, USA, Supplementary Table S1), for 23 weeks. All mice were caged two by two at 20-24 °C, humidity 55% ± 10% with a strict 12 h light cycle. Body weight was recorded weekly and food intake per cage was recorded twice a week. One mouse died during cheek blood sampling at Week 9.

Sampling

The mice were transferred to clean cages the day before feces sampling. From each cage, around 50-100 fecal pellets from the two mice were collected for microbiota analysis. In the meantime, the two mice were kept in two clean cages until defecation, and fecal pellets were collected immediately after defecation for short chain fatty acids (SCFAs) analysis.

Urine samples were collected at the terminal week (Week 23). Blood was sampled from the cheek into EDTA-coated micro tubes for flow cytometry analysis at Week 0, 9 and 23. At Week 22, fasting blood from the cheek was sampled into EDTA-coated tubes and centrifuged (2000 g, 5 min) to obtain plasma for insulin analysis before the oral glucose tolerance test (OGTT). All samples were kept on ice until transfer to -80 °C.

At the terminal week, one mouse from each cage was fasted for 6 hours after onset of the light cycle, while the other one was not fasted. (Fasting was carried out to allow measurements of intestinal permeability, which failed and are therefore not included). All mice were anesthetized with a Hypnorm/Dormicum mixture (0.315 mg/mL Fentanyl, 10 mg/mL Fluanisone and 5 mg/mL Midazolam) injected subcutaneously as a 1:1:2 water solution (0.006 mL/g body weight). Blood was sampled from the periorbital plexus into EDTA-coated tubes and centrifuged (2000 g, 5 min) to obtain plasma. The mice were then euthanized by cervical dislocation and dissected. Liver and epididymal white adipose tissue (eWAT) were weighed. Samples from ileum, colon and liver were placed in the RNA*later* RNA stabilization reagent (QIAGEN, Hilden, Germany) overnight; liver samples were flash-frozen in liquid nitrogen for triglyceride analysis and eWAT samples were flash-frozen for RNA extraction and additional samples of liver and eWAT were placed in 4% paraformaldehyde for histological analyses. Luminal contents from ileum, cecum and colon were flash-frozen. All samples except the paraformaldehyde fixed tissues were stored at -80 °C until further analyses.

Biochemical Measurements in blood and plasma

HbA1c was measured in tail vein blood using a DCA Vantage Analyzer (Siemens, Erlangen, Germany). OGTT was performed by oral administration of 75 mg glucose in 0.15 mL solution after 6 hours of fasting. Glucose in the tail vein blood was measured before glucose dosage and at 15, 30, 60, 90, 120 and 180 minutes after dosage using a FreeStyle Lite glucometer (Abbott Diabetes Care Inc., Berkshire, UK).

Plasma insulin was measured using a Mouse Insulin ELISA Kit (Mercodia, Uppsala, Sweden). HOMA-IR was calculated as insulin [mU/L]*glucose [mmol/L])/22.5. Plasma alanine aminotransferase was measured with an ELISA kit (MyBioSource, San Diego, CA, USA). Plasma cytokines, IL-1β, IL-6, IFN-γ, TNF-α and IL-10, were measured using a custom V-PLEX Mouse Biomarkers ELISA Kit (Meso Scale Discovery, Rockville, MD, USA).

SCFA analysis

SCFAs were analyzed in cecal samples and feces by Gas Chromatography Mass Spectrometry essentially as previously described [12,13].

Frozen cecum content and fecal pellets were thawed on ice. Cecum contents (5-25 mg) were homogenized in 250 µl methanol, 250 µL Milli-Q water and 10 µL internal standard (100 mM 2-ethylbutyric acid in 12% formic acid, Sigma-Aldrich, St.Louis, MO, USA) using a micro-homogenizer. Similarly, one or two fecal pellets per sample were homogenized in 1.5 mL water and 100 µl internal standard using a bead-beater, and incubated for 10 min at room-temperature with slow shaking. Acidity of samples was adjusted to pH = 2-3 using 3M HCl. The samples were then centrifuged at 10,000 g for 10 min, and supernatants were filtered through 0.45 µm Phenex-NY syringe filters (Phenomenex, Værløse, Denmark). External calibration was performed using standard solution mixtures of acetic acid, propionic acid, butyric acid, iso-butyric acid, valeric acid, iso-valeric acid, caproic acid and 2-ethylbutyric acid (Sigma-Aldrich) in the concentrations 10, 20, 50, 100, 250, 500 and 1,000 mM with extra acetic acid, propionic acid and butyric acid in the concentrations 2000 and 5000 mM.

Aliquots (3 µL) of each sample were injected into a HP 6890 GC system (Agilent Technologies, Santa Clara, CA, USA) with a CP-FFA WCOT fused silica capillary column (25 m x 0.53 mm i.d. coated with 1 µm film thickness, Chrompack, EA Middelburg, The Netherlands). The carrier gas was helium at a flow rate of 20 mL/min. The initial oven temperature of 60 °C was maintained for 0.25 min, raised to 180 °C at 8 °C/min and held for 3 min, then increased to 215 °C at 20 °C/min, and finally held at 215 °C for 5 min. The temperature of the front inlet detector and the injector was 250 °C. The flow rates of hydrogen, air and helium as makeup gas were 40, 450, and 45 mL/min, respectively. The run time for each analysis was 22 min. Data handling was performed using the OpenLAB Chromatography Data System ChemStation Edition software (Rev.A.10.02). The concentration of SCFA in the samples was calculated against the individual external standards, and adjusted according to the loss of internal standard.

Urine metabolome profiling with Ultra Performance Liquid Chromatography Mass Spectrometry (UPLC-MS)

Frozen urine samples were thawed on ice and centrifuged at 10,000 g for 10 min at 4 °C to remove particles. Subsequently, samples were diluted 1:100 with Milli-Q water, mixed by vortexing, and pipetted into LC vials. Pooled quality control (QC) samples were prepared by mixing aliquots of all urine samples, thereby ensuring the QC sample represented the whole sample set. The samples were kept on ice during preparation.

The column was conditioned by running a couple of blank samples (water) followed by five injections of the QC sample before urine samples were injected. The QC sample was analysed once for every ten urine samples throughout the LC-MS analysis to provide data from which the reproducibility could be assessed. For each sample, 2 µL of diluted urine (1:100 in water) was analysed in both negative and positive mode by a UPLC-QTOF-MS system consisting of Dionex Ultimate 3000 RS liquid chromatograph (Thermo Scientific, Sunnyvale, CA, USA) coupled to a Bruker maXis time of flight mass spectrometer equipped with an electrospray interphase (Bruker Daltonics, Bremen, Germany). The analytes were separated on a Poroshell 120 SB-C18 column with a dimension of 2.1 x 100 mm and 2.7 µm particle size (Agilent Technologies, CA, USA) based on the settings according to Want et al. [14]. Shortly, the column was held at 40 °C and the sampler at 4 °C. The UPLC mobile phases consisted of 0.1% formic acid in water (solution A) and 0.1% of formic acid in acetonitrile (solution B). While containing a constant flow rate of 0.4 mL/min, the analytes were eluted using the following gradient. Solvent programming was isocratic 1% B for 1 min followed by a linear gradient up to 15% at 3 min, then a linear gradient up to 50% B at 6 min, and finally a linear gradient up to 95% B at 9 min. The final gradient composition, 95% B, was held constant until 10 min, followed by a return of the solvent composition to initial conditions at 10.1 min and equilibration until 13 min. Mass spectrometry data were collected in full scan mode at 2 Hz with a scan range of 50-1000 mass/charge (m/z).

The following electrospray interphase settings were used: nebulizer pressure 2 bar, drying gas 10 L/min, 200 °C, capillary voltage 4500 V. For MS/MS analyses, a ramp collision energy ranging from 10 to 30 eV was applied on a scheduled precursor list. To improve the measurement accuracy, external and internal calibrations were done using sodium formate clusters (Sigma-Aldrich) and in addition a lock-mass calibration was applied (hexakis(1H,1H,2H-perfluoroetoxy)phosphazene, Apollo Scientific, Manchester, UK).

The raw LC-MS data were converted to mzXML files using Bruker Compass DataAnalysis 4.2 software (Bruker Daltonics) and were then pre-processed through noise filtering, peak detection, and alignment using the open-source R package XCMS (v1.38.0) [15]. Noise filtering settings included that features should be detected in minimum 50 % of samples within a group. Data tables were generated comprising m/z, retention time, and intensity (peak area) for each variable in every sample. For each sample, the urinary features were normalized to the total intensity of the given sample. Subsequently, the data were filtered using the pooled QC samples: features with coefficient of variation above 30% in the QC samples were excluded, and features with a retention time above 8 min were also excluded. The resultant data was imported into the LatentiX 2.12 software (Latent5; http://www.latentix.com) for further multivariate analysis. Unsupervised principal component analysis (PCA) was performed on mean-centered data to visualize general clustering trends. Discriminating features were evaluated by an unpaired t test with Welch's correction followed by the Benjamini-Hochberg false discovery rate (FDR) correction [16] with a significance level of 0.05 (q value). Metabolite candidates were identified by searching the accurate masses of parent ions and fragments (from MS/MS), against the METLIN [17] and HMDB databases [18]. The metabolites were identified according to the four different levels described by the Metabolomics Standard Initiative [19]. To confirm the identity of dopamine-glucuronide, a urine sample was diluted in 0.1 M sodium phosphate buffer (pH = 7.4) and incubated with β-glucuronidase from *E. coli* K12 (Roche Diagnostics GmbH) at 37 °C for 1-2 hours. Subsequently, the urine sample was injected onto the UPLC-MS for analysis, which allowed the deglucuronidated dopamine *m/z* feature to be compared with the authentic standard of dopamine, which was obtained from Sigma-Aldrich (Schnelldorf, Germany). A heat-map showing the log-transformed relative abundance of the discriminating metabolites was generated by the Heatmap function of MetaboAnalyst [20] using Euclidean distance measure and the ward clustering algorithm.

16s rRNA gene sequencing

Bacterial DNA was extracted from feces and luminal contents using the PowerLyzer PowerSoil DNA Isolation Kit (Mo Bio Laboratories, Carlsbad, CA, USA). Around 10-20 fecal pellets (100 mg) per sample were used to make sure that both mice in the cage were represented. DNA concentrations were measured using the Nanodrop Spectrophotometer ND-1000 (Thermo Scientific, Wilmington, DE, USA). Variable Region 3 (V3) of the 16S rRNA gene was amplified using a universal forward primer with a unique 10-12 bp barcode (IonXpress barcode, Ion Torrent) for each bacterial community (PBU 5'-A-adapter-TCAG-barcode-GAT-CCTACGGGAGGCAGCAG-3') and a universal reverse primer (PBR 5'-trP1-adapter-ATTACCGCGGCTGCTGG-3'). The PCR reactions were conducted with 5 ng template DNA, 10 mmol/L dNTP, 1 µmol/L forward/reverse primer, 4 µL HF-buffer and 0.2 µL Phusion High-Fidelity DNA polymerase (Thermo Scientific, Vilnius, Lithuania) in a total reaction volume of 20 µL. PCR conditions were 30 s at 98 °C, 24 cycles of 15 s at 98 °C, 30 s at 72 °C, followed by 5 min at 72 °C. For a few samples that did not yield sufficient PCR products, an initial PCR was performed with universal primers without barcodes or adaptors (30 cycles) followed by a second round of PCR (15 cycles) with barcoded primers using a 10-fold dilution of the first PCR product as template. PCR products were separated on a 1.5% agarose gel at 3.5 V/cm for 90 minutes, and bands of the expected size (approximately 260 bp) were excised from the gel. DNA was purified from the excised gel using the MinElute Gel Extraction Kit (Qiagen, Germany) according to the instructions of the manufacturer. DNA concentrations were determined using the Qubit 2.0 fluorometer (Invitrogen, Carlsbad, CA, USA) with the dsDNA HS assay (Invitrogen, Eugene, OR, USA), and equal amounts of PCR products from each community were pooled to construct a library. Sequencing was performed using the Ion PGM Template OT2 200 Kit and the Ion PGM Sequencing 200 Kit v2 with the 318-chip (Ion Torrent).

Sequencing data analysis
Sequences were de-multiplexed, trimmed to eliminate primers, and filtered with a length range of 125-180 bp using the CLC Genomic Workbench v7.0.3 (Qiagen, Aarhus, Denmark). Each sequence was classified to the lowest possible taxonomic rank (assignment confidence ≥ 50%) using the Ribosomal Database Project (RDP) Classifier v2.10.1 [21], and collapsed at genus, family, phylum and domain levels. The depths (range, median) of the resulting phylotype data table were: 35,156-95,090, 59,500 for fecal samples, 35,672-93,032, 73,251 for ileal samples, 23,216-57,266, 42,276 for cecal samples and 25,066-52,617, 42,754 for colonic samples.

Operational taxonomic units (OTUs) were generated using UPARSE v8.0.1623 [22]. All sequences were subjected to quality filtering with a cut-off of maxee of 3.5 (discard reads with > 3.5 total expected errors for all bases in the read). Unique sequences except singletons were clustered at 97% sequence homology. Chimeras were first filtered by the UPARSE-OTU algorithm and then by the UCHIME algorithm [23] using the RDP classifier training database v9 and the default threshold. Taxonomies of OTU representative sequences were also assigned using the RDP classifier. The depths (range, median) of the resulting OTU data table were: 28,606-74,454, 47230 for fecal samples, 30,421-89,534, 67,731 for ileal samples, 14,542-41,654, 29,559 for cecal samples and 15,495-37,974, 29,731 for colonic samples.

Microbiota α diversity (Shannon index) and β diversity were analyzed using Qiime 1.8.0 [24]. OTU representative sequences were pooled with a 16S rRNA gene sequence assigned as *Methanosarcina* within the Archaea, and aligned to the Greengenes core set (Greengenes 13_5 PyNAST aligned 85% OTU representative sequences) [25] using PyNAST [26]. A phylogenetic tree was created using FastTree [27]. Using Dendroscope v3.3.2, the tree was re-rooted with the Archaea outgroup, and then the outgroup was pruned from the tree, thereby generating a phylogenetic tree for downstream analyses. The Principle coordinate analyses (PCoA) was performed on UniFrac distances [28] between microbial communities, and the OTU tables were rarefied to the lowest number of reads in each PCoA model. The ADONIS test was performed to assess the differential clustering of microbial communities using the *vegan* R package v2.3-0 [29], and feeding status (fasted/non-fasted) was included as a variable when the ileal, cecal and colonic samples were analysed.

To discover features, i.e. OTUs and bacterial groups at genus/family/phylum levels (based on the phylotype data table), that were differentially abundant in the Gliadin- and Gliadin+ mice, features that were less abundant than 0.02% of average numbers of total bacteria in both groups and features that presented in less than 50% of samples in both groups were filtered out. The matrices of relative abundances were permutated 10,000 times, and p values represent fraction of times that permuted differences assessed by Welch's t test were greater than or equal to real differences, followed by the FDR correction.

Gene expression analysis using real time RT-PCR
Total RNA from liver, ileal and colonic tissues was extracted using the RNeasy Plus Mini Kit (Qiagen, Germany) according to the product protocol, while for adipose tissue, total RNA was harvested by trizol and chloroform based purification followed by kit extraction. For gut barrier function related genes, RNA concentration and purity were determined using the Nanodrop Spectrophotometer ND-1000, and cDNA was synthesized from 1.4 µg RNA in 20 µL reactions using the SuperScript VILO cDNA Synthesis Kit (Invitrogen, Carlsbad, CA, USA), and diluted 20-fold for further use. Quantitative real time RT-PCR was performed with a SYBR Green I Master (Roche Diagnostics GmbH, Mannheim, Germany) and a LightCycler 480 system (Roche Diagnostics GmbH) using the described primer sets (Supplementary Table S5), and the PCR reactions were run under the following conditions: 95 °C for 5 min; 40 cycles of 95 °C for 10 sec, 55 °C for 10 sec, and 72 °C for 30 sec; melting curve generation with preparation with 95 °C for 5 sec, 65 °C for 1min and increasing the temperature to 98 °C with a rate of 0.11 °C/sec with continuous fluorescence detection. The amplification efficiency of each primer was assessed by a standard curve. The amount of each mRNA was normalized to the geometric mean of expression levels of phosphoglycerate kinase 1 gene

(*Pgk1*), actin beta gene (*Actb*) and glyceraldehyde 3-phosphate dehydrogenase gene (*Gapdh*). For the remaining analyses, RNA concentrations were measured using a Qbit 2.0 fluorometer, and cDNA was synthesized by a High Capacity cDNA Reverse Transcription Kit (Applied Biosystems) from 2 µg RNA. Real time PCR of the cDNA was performed with a TaqMan Fast Universal PCR Master Mix (Applied Biosystems, Foster city, CA, USA) and a 7900HT Fast Real-time PCR system (Applied Biosystems) using primers and probes (Supplementary Table S6) purchased from Integrated DNA Technologies (Leuven, Belgium). The PCR reactions were run under the following conditions: 95 °C for 20 sec; 40 cycles of 95 °C for 1 sec and 60 °C for 20 sec. Normalization was done with the beta-2 microglobulin gene (*B2m*) and *Gapdh*.

Flow cytometry

Blood samples were lysed in a red blood cell lysis buffer (ACK buffer, 154.95 mM ammonium chloride, 9.99 mM sodium hydrogen carbonate, 0.0995 mM Disodium EDTA, in PBS) for 5 minutes at room temperature. Dissected and cleaned adipose tissue was homogenized by 40 minutes incubation at 37 °C with collagenase II (Sigma-Aldrich C6885), while liver tissue was cleaned and manually grinded with a piston. Homogenized liver and adipose tissues were filtered using a BD Falcon cell strainer (70 µm filter, BD Bioscience, Bedford, MA, USA) and washed in PBS with 1% FCS and 2 µM monensin (Sigma-Aldrich). Dissected lymphoid tissues (MLN and PP) were washed in PBS (without Ca^{2+} and Mg^{2+}) with 1% FCS, 2 µM monensin (Sigma-Aldrich) and 3 mM EDTA and filtered in a 70 µm filter. All single cell suspensions were counted on a Nucleocounter NC-100 (ChemoMetec, Denmark) and plated.

All cell suspensions were Fc-blocked (CD16/CD32, BD Biosciences) and surface stained in PBS with 1% fetal calf serum and 2 µM monensin and subsequently fixed and permeabilized for 20 min at 4 °C (Cytofix/Cytoperm, BD Biosciences) followed by intracellular staining. Samples were washed and re-suspended in PBS with 1% fetal calf serum and 0.1% sodium azide, and Count Bright beads were added (Invitrogen Molecular Probes) according to manufacturer's instructions. Samples were run on a BD FACSCanto II (BD Biosciences) flow cytometer. Data were analysed using Flowjo software (V10.0.7, Treestar). Gating strategies are shown in S5 Fig and S6 Fig.

Antibodies for surface and intracellular staining included CD45-APC-Cy7 (BD, 30-F11), CD8-FITC (BD, 53-6.7), CD3 (BD, 500A2), NK1.1-FITC (BD, PK136), B220-V500 (BD, RA3-6B2), Siglec-F-PE (BD, E50-2440), cKit-V450 (BD, 2B8), CD11c-PE-Cy7 (BD, HL3), Arg1-Alexa Fluor 647 (BD, 19/Arginase-1), CD103-PE (BD, M290), Ly6G-PE-Cy7 (BD, 1A8), TCRδ-BV421 (BD, GL3), CD11b-V500 (BD, M1/70), IFN-γ-PerCP-Cy5.5 (BD, XMG1.2), FoxP3-Alexa Fluor 647 (BD, MF23), TNFα-PE (BD, MP6-XT22), CD4-PE-Cy7 (eBioscience, GK1.5), CD1d-PE (eBioscience, 1B1), CD19-PerCP-Cy5.5 (eBioscience, 1D3), IgD-eFluor 450 (eBioscience, 11-26c), F4/80-FITC (eBioscience, BM8), Ly6C-PerCP-Cy5.5 (eBioscience, HK1.4), IgM-PE-Cy7 (eBioscicence, II/41), IL-4-APC (eBioscience, 11B11), IL-17A-PE (eBioscience, 17B7), IL-12p35-eFluor 660 (eBioscience, 4D10p35), Siglec-H-PerCP-eFluor 710 (eBioscience, 440c), NKp46-PE-Cy7 (29A1.4), CD49b-FITC (eBioscience, HMa2), FcεRIα-PerCPeFluor 710 (eBioscience, MAR-1), IL-10-APC (eBioscience, JES5-16E3), CD5-FITC (eBioscience, 53-7.3), CD115-APC (eBioscience, AFS98), IL-4-PE (eBioscience, 11B11), CX3CR1-Pacific Blue (eBioscience, polyclonal) and CCR2-PE (R&D, 475301).

Histology

Liver and adipose tissues were fixed for 24 h in 4% paraformaldehyde (Sarstedt, 51.1703.009) and then kept in PBS for 21 days until dehydration in ethanol followed by paraffin embedding. Tissues were sectioned and stained with hematoxylin and eosin stain (Sigma Aldrich, St. Louis, MO, USA). Liver steatosis was initially analyzed by automated quantification of lipid droplets (ImageJ, v1.49). This was followed by a second evaluation by an independent lab using a non-alcoholic steatohepatitis grading score of steatosis, inflammation and hepatocellular ballooning. For the adipose tissue, adipocytes were measured manually in four independent tissue sections per tissue sample, and an average of 150 cells were quantified per section.

In order to confirm our observations, adipocyte sizes were also measured blindly by an independent lab using ImageJ.

SUPPLEMENTAL REFERENCES

1. MS, B. *et al.* CD73 is critical for the resolution of murine colonic inflammation. *J Biomed Biotechnol* 260983 (2012).
2. Liu, G. *et al.* miR-147, a microRNA that is induced upon Toll-like receptor stimulation, regulates murine macrophage inflammatory responses. *Proc. Natl. Acad. Sci. U. S. A.* **106,** 15819–24 (2009).
3. Bergström, A. *et al.* Nature of bacterial colonization influences transcription of mucin genes in mice during the first week of life. *BMC Res. Notes* **5,** 402 (2012).
4. Acharya, P. *et al.* Distribution of the tight junction proteins ZO-1, occludin, and claudin-4, -8, and -12 in bladder epithelium. *Am. J. Physiol. Renal Physiol.* **287,** F305-18 (2004).
5. Corridoni, D. *et al.* Probiotic bacteria regulate intestinal epithelial permeability in experimental ileitis by a TNF-dependent mechanism. *PLoS One* **7,** e42067 (2012).
6. Reynolds, J. M. *et al.* Cutting edge: regulation of intestinal inflammation and barrier function by IL-17C. *J. Immunol.* **189,** 4226–30 (2012).
7. Becker, S. *et al.* Bacteria regulate intestinal epithelial cell differentiation factors both in vitro and in vivo. *PLoS One* **8,** e55620 (2013).
8. Hoebler, C., Gaudier, E., De Coppet, P., Rival, M. & Cherbut, C. MUC genes are differently expressed during onset and maintenance of inflammation in dextran sodium sulfate-treated mice. *Dig. Dis. Sci.* **51,** 381–9 (2006).
9. Hwang, I. *et al.* Alteration of tight junction gene expression by calcium- and vitamin D-deficient diet in the duodenum of calbindin-null mice. *Int. J. Mol. Sci.* **14,** 22997–3010 (2013).
10. Bull-Otterson, L. *et al.* Metagenomic analyses of alcohol induced pathogenic alterations in the intestinal microbiome and the effect of Lactobacillus rhamnosus GG treatment. *PLoS One* **8,** e53028 (2013).
11. Kristensen, M. B. *et al.* Neonatal microbial colonization in mice promotes prolonged dominance of CD11b(+)Gr-1(+) cells and accelerated establishment of the CD4(+) T cell population in the spleen. *Immunity, Inflamm. Dis.* **3,** 309–20 (2015).
12. Zhao, G., Nyman, M. & Jönsson, J. A. Rapid determination of short-chain fatty acids in colonic contents and faeces of humans and rats by acidified water-extraction and direct-injection gas chromatography. *Biomed. Chromatogr.* **20,** 674–82 (2006).
13. Nejrup, R. G. *et al.* Lipid hydrolysis products affect the composition of infant gut microbial communities in vitro. *Br. J. Nutr.* **114,** 63–74 (2015).
14. Want, E. J. *et al.* Global metabolic profiling procedures for urine using UPLC-MS. *Nat. Protoc.* **5,** 1005–1018 (2010).
15. Smith, C. A., Want, E. J., O'Maille, G., Abagyan, R. & Siuzdak, G. XCMS: Processing Mass Spectrometry Data for Metabolite Profiling Using Nonlinear Peak Alignment, Matching, and Identification. *Anal. Chem.* **78,** 779–787 (2006).
16. Benjamini, Y. & Hochberg, Y. Controlling the False Discovery Rate - a Practical and Powerful Approach to Multiple Testing. *J. R. Stat. Soc. Ser. B-Methodological* **57,** 289–300 (1995).
17. Smith, C. A. *et al.* METLIN: a metabolite mass spectral database. *Ther. Drug Monit.* **27,** 747–51 (2005).

18. Wishart, D. S. *et al.* HMDB 3.0--The Human Metabolome Database in 2013. *Nucleic Acids Res.* **41,** D801-7 (2013).

19. Sumner, L. W. *et al.* Proposed minimum reporting standards for chemical analysis Chemical Analysis Working Group (CAWG) Metabolomics Standards Initiative (MSI). *Metabolomics* **3,** 211–221 (2007).

20. Xia, J. & Wishart, D. S. Web-based inference of biological patterns, functions and pathways from metabolomic data using MetaboAnalyst. *Nat. Protoc.* **6,** 743–60 (2011).

21. Wang, Q., Garrity, G. M., Tiedje, J. M. & Cole, J. R. Naive Bayesian classifier for rapid assignment of rRNA sequences into the new bacterial taxonomy. *Appl. Environ. Microbiol.* **73,** 5261–7 (2007).

22. Edgar, R. C. UPARSE: highly accurate OTU sequences from microbial amplicon reads. *Nat. Methods* **10,** 996–8 (2013).

23. Edgar, R. C., Haas, B. J., Clemente, J. C., Quince, C. & Knight, R. UCHIME improves sensitivity and speed of chimera detection. *Bioinformatics* **27,** 2194–200 (2011).

24. Caporaso, J. G. *et al.* QIIME allows analysis of high-throughput community sequencing data. *Nat. Methods* **7,** 335–6 (2010).

25. McDonald, D. *et al.* An improved Greengenes taxonomy with explicit ranks for ecological and evolutionary analyses of bacteria and archaea. *ISME J.* **6,** 610–8 (2012).

26. Caporaso, J. G. *et al.* PyNAST: a flexible tool for aligning sequences to a template alignment. *Bioinformatics* **26,** 266–7 (2010).

27. Price, M. N., Dehal, P. S. & Arkin, A. P. FastTree: computing large minimum evolution trees with profiles instead of a distance matrix. *Mol. Biol. Evol.* **26,** 1641–50 (2009).

28. Lozupone, C. & Knight, R. UniFrac: a new phylogenetic method for comparing microbial communities. *Appl. Environ. Microbiol.* **71,** 8228–35 (2005).

29. Oksanen, J. *et al.* vegan: Community Ecology Package. (2015).
